# Comprehensive review of dynamical simulation models of packed-bed systems for thermal energy storage applications in renewable power production

**DOI:** 10.1016/j.heliyon.2025.e42803

**Published:** 2025-02-20

**Authors:** D. Pérez-Gallego, J. Gonzalez-Ayala, A. Medina, A. Calvo Hernández

**Affiliations:** a Universidad de Salamanca, Department of Applied Physics, Salamanca, 37008, Castilla y León, Spain; b Universidad de Salamanca, Institute of Physics and Mathematics (IUFFYM), Salamanca, 37008, Castilla y León, Spain

**Keywords:** Energy storage technologies, Thermal energy storage, Packed-bed systems, Thermoclines numerical simulation, Efficient computing, Charge-discharge efficiencies

## Abstract

The need for large-scale energy storage in the context of renewable electricity production worldwide is evident. Among the various energy storage methods, thermal energy storage stands out. It is independent of geographical location, allows high storage capacities, does not require scarce materials, and is cheaper than its direct competitors. Currently, several technologies are being intensively developed. In some of them, packed-bed systems play a central role: a heat transfer fluid heats up or releases heat from a porous solid that acts as a thermal energy reservoir. This work compiles their application to concepts such as concentrated solar power, pumped thermal energy storage, and compressed or liquid air energy storage. Different physical models with diverse refinement degrees and the corresponding computational schemes are comprehensively presented. Comparison with previous experimental works includes gas or liquid heat transfer fluids, sensible or latent heat transfers, and a wide range of temperature levels. It is shown that the continuous 1D solid phase model solved with an implicit Euler method provides satisfactory results with a reasonable computing time for various systems. The influence of time step and spatial mesh is surveyed, as well as that of pressure drops. Efficiencies and stored energies are calculated for some particular cases, and sensitivity analysis is presented, including parameters such as fluid velocity in discharge and storage time. Concerning the latter, discharge efficiencies for long-time storage (between 10 and 15 h) are fairly good, between 0.39 and 0.20.

## Highlights

• Applications of packed-bed thermal storage for renewable power production (solar and wind).
• Assessment of physical models, computational methods, and useful tips for modeling packed-bed systems for new applications.
• Liquid or gas heat transfer fluids, sensible and latent materials.
• Estimations of computing times for different time steps and spatial mesh for several numerical approaches.
• Analysis of performance and energy losses with storage time.

## Graphical abstract

**Figure:**
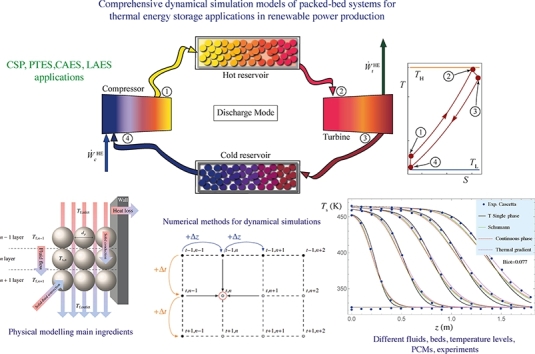

## Nomenclature

ab: Ratio between surface and volume of tank $\left({\text{m}}^{-1}\right)$
as: Surface area of particles per unit tank volume $\left({\text{m}}^{-1}\right)$
A: Area of the particles (${\text{m}}^{2}$)
Bi: Biot number
cp: Specific heat capacity (J/(kg⋅K))
dp: Diameter of bed particles (m)
D: Diameter of bed (m)
e: Thickness of wall or insulator (m)
E: Energy (J)
g: Gravity acceleration $\left(\text{m}/{\text{s}}^{2}\right)$
Gr: Grashof number
h: Convection coefficient $\left(\text{W}/\left({\text{m}}^{2}\cdot \text{K}\right)\right)$
$h_{\text{eff}}$: Effective convection coefficient $\left(\text{W}/\left({\text{m}}^{2}\cdot \text{K}\right)\right)$
K: Thermal conductivity (W/(m⋅K))
L: Tank length (m)
m˙: Mass flux (kg/s)
Nt: Total number of temporal points
Nu: Nusselt number
Nz: Total number of spatial points
P: Pressure (Pa)
Pr: Prandtl number
Q: Volumetric flux $\left({\text{m}}^{3}/\text{s}\right)$
r: Radius (m)
Rep: Reynolds number
Ra: Rayleigh number
t: Time (s)
t¯: Dimensionless time
T: Absolute temperature (K)
u: Interstitial velocity ($\text{m}/{\text{s}}^{2}$)
Us: Superficial velocity ($\text{m}/{\text{s}}^{2}$)
U: Effective heat loss coefficient $\left(\text{W}/\left({\text{m}}^{2}\cdot \text{K}\right)\right)$
V: Packed-bed Volume $\left({\text{m}}^{3}\right)$
z¯: Dimensionless length

**Greek letters**
ϵ: Void fraction
εmat: Emissivity
η: Radial component of particles
ηtotal: Efficiency
θ: Dimensionless temperature
μ: Dynamic viscosity (Pa⋅s)
ν: Kinematic viscosity $\left(\text{m}/{\text{s}}^{2}\right)$
ρ: Mass density $\left(\text{kg}/{\text{m}}^{3}\right)$
σ: Stefan-Boltzmann constant $\left(\text{W}/\left({\text{m}}^{2}\cdot \text{K}\right)\right)$
τ: Time lag (s)

**Subscripts**
∞: Ambient temperature
eff: Effective
ext: External
f: Heat transfer fluid
*in*: Internal
ins: Packed-bed insulator
*mat*: Material
max: Maximum
min: Minimum
r: Radial
*rad*: Radiation
s: Storage material
t: Total
w: Packed-bed wall
z: Axial

**Acronyms**
CAES: Compressed Air Energy Storage
A-CAES: Adabatic-CAES
AA-CAES: Advanced Adiabatic-CAES
I-CAES: Isothermal-CAES
CSP: Concentrated Solar Power
EES: Electric Energy Storage
FDM: Finite Difference Method
HTF: Heat Transfer Fluid
LAES: Liquid Air Energy Storage
LCA: Life Cycle Assessment
LCoE/S: Levelized Cost of Electricity/Storage
PB: Packed-Bed
PCM: Phase Change Materials
PTES: Pumped Thermal Energy Storage
TES: Thermal Energy Storage
TRL: Technology Readiness Level

## 1 Introduction

Electricity produced by intermittent renewable energy sources is increasing rapidly worldwide. The development of novel clean energy technologies is nowadays essential because of the necessity to limit harmful and greenhouse emissions, the depletion of fossil fuels, price variability, and the increasing electric energy demand. Moreover, the ongoing efforts to electrify the transport sector require accelerating the deployment of renewable technologies. Nevertheless, the irregular nature of solar and wind resources makes the dispatchable on-demand production of clean energy difficult. Thus, low-cost and efficient electrical energy storage is essential to balance the mismatch between energy supply and demand. So, an open challenge of the energy transition for the following years is to develop technological routes to efficiently produce electric energy in a renewable and clean way, ensuring production control and dispatchability [1]. Apart from initiatives such as the enhancement of grid interconnections or over-sizing renewable capacity, this is associated with large-scale energy storage [2].

Several storage technologies at the MW scale are at dissimilar readiness levels. A possible way to classify energy storage technologies, as proposed by Rahman et al. [3] encompasses:

• Mechanical: including pumped hydro, flywheels, compressed air storage (CAES, A-CAES, etc.) [4], and liquid air energy storage (LAES) [5].
• Chemical: hydrogen and synthetic fuels [6].
• Electrical: supercapacitors.
• Electrochemical: including secondary (PbA, Na-S, Li-ion, Ni-Cd, ...) and flow (vanadium redox and Zn-Br batteries).
• Thermal: sensible, latent heat, and thermo-chemical [7]. Some technologies within CAES also can be considered as belonging to this category, as it will be commented on later, because they have mechanical and thermal elements. Pumped thermal energy storage (PTES) [8] and Concentrating Solar Power (CSP) [9] can also be included here. Details on both will be exposed later in this Introduction.

This classification should not be considered rigid but just as a simple reference guide because some technologies involve different cross-concepts. Nowadays, pumped hydro energy storage by large constitutes the most deployed technology, representing more than 90% of the total grid-scale storage [10]. Still it is constrained to regions with particular hydrological, meteorological, and seasonal conditions.

Among the basic characteristics that storage technologies should fulfill, the following are crucial: large-scale capacity, high energy density, good round-trip efficiencies, flexible storing periods, possibility to use environment-friendly non-critical materials, a large number of operation cycles, independence of location conditions, and economic affordability for investors and stakeholders. Gallo et al. compile in [11] in a very accessible way several possible applications of large-scale storage and the particular technical requirements for each of those applications. Rahman et al. [3] discuss, apart from possible future cost estimates, life cycle assessment (LCA) studies of several storage technologies.

Thermal energy storage (TES) techniques have advantages that make them promising in the short- and mid-term. Two of them are remarkable: it benefits from relatively high energy densities (that should translate into a low cost per MWh of storage capacity) and its independence from geographical constraints [13] (except for CAES). In the next few years, TES can play a significant role in large-scale renewable power production because of its direct integration in concentrated solar, wind, and photovoltaic technologies. Recent reviews on these concepts for different applications and their development stage are due to McTigue et al. [2], [14] and Khan et al. [15]. As defined by IRENA [12], TES consists of storing energy in thermal form using a storage medium. Stored energy could be used later for different purposes, such as power generation, heating, or cooling. Among its applications, IRENA [12] mention the following ones: demand shifting, variable supply integration, sector integration, seasonal storage, and network reinforcement deferral. Table 1 summarizes data from IRENA [12] the present and predicted mid-term characteristics of the main types of TES technologies with CSP, and Table 2 shows the corresponding data for PV and wind generation. In 2018, the variable renewable energy (VRE) share in power generation was 10%, and 35% is estimated for 2030, and 60% of global power generation is expected by 2050. Thus, the growth of TES will be pivotal in such scenarios. In 2019, most of TES installed capacity was focused on heating applications, and only 10% devoted to power production, but 50% is foreseen for 2050.

**Table 1:**
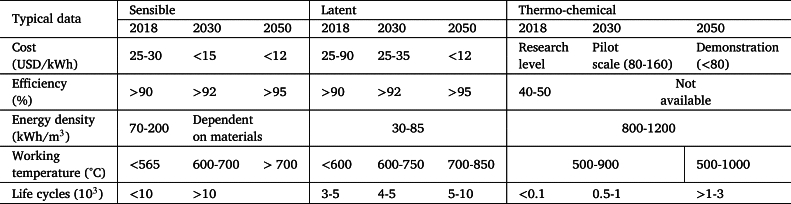
Present and predicted mid- and long-term foreseen outputs for the main groups of TES technologies for CSP applications [12].

**Table 2:**
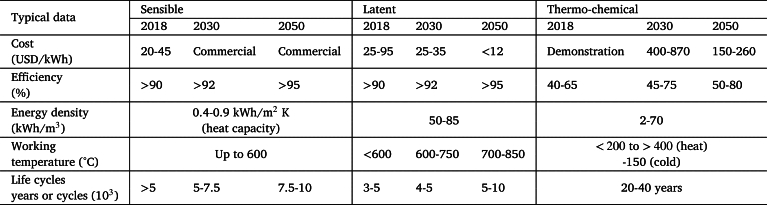
Present and predicted mid- and long-term foreseen outputs for the main groups of TES technologies for PV and wind generation [12].

### 1.1 Packed-bed (PB) main ingredients

In any TES technology, at least three main elements, usually coupled subsystems, are incorporated. The first one operates during the charge step, transforming the input energy (for instance, electrical energy for TES systems coupled to wind or photovoltaic installations or direct thermal energy for CSP plants) into storable thermal energy. Second, a system capable of storing or retaining the thermal energy for the target period, and third, another subsystem responsible for converting, when required, the stored thermal energy back to electric energy (discharge process).

Among the systems capable of collecting, storing, and releasing thermal energy, PBs have received extensive interest for a long ago. From practical viewpoints because of their broad applicability (particular technologies and temperature levels), economic reasons, and environmental advantages (storing materials are not scarce and even can be taken from plants' surroundings to profit location possibilities). From a purely scientific viewpoint, because of the complexity of the analysis of these systems and the challenges it entails, physical models incorporate realistic enough ingredients for each particular application and the numerical (and computational) methods involved. There are ancient papers in the literature that are still cited nowadays, like the pioneering work by Schumann in 1929 [17], and there are also reviews on the field from 30 years ago, as the also very frequently cited work by Al-Nimr et al. [18]. As these authors mention, PBs are a particular application of thermal processes involving fluid motion through porous media.

Over the years, their applications have changed and adapted to technological evolution. Hence, the utilization of PB nowadays is probably quite different from what it was some time ago. Remarkably, there is an extensive interest in studying its applications to the storage of energy produced through renewable resources such as wind and solar. Thus, the liveliness of this field is plentiful nowadays.

**Figure 1:**
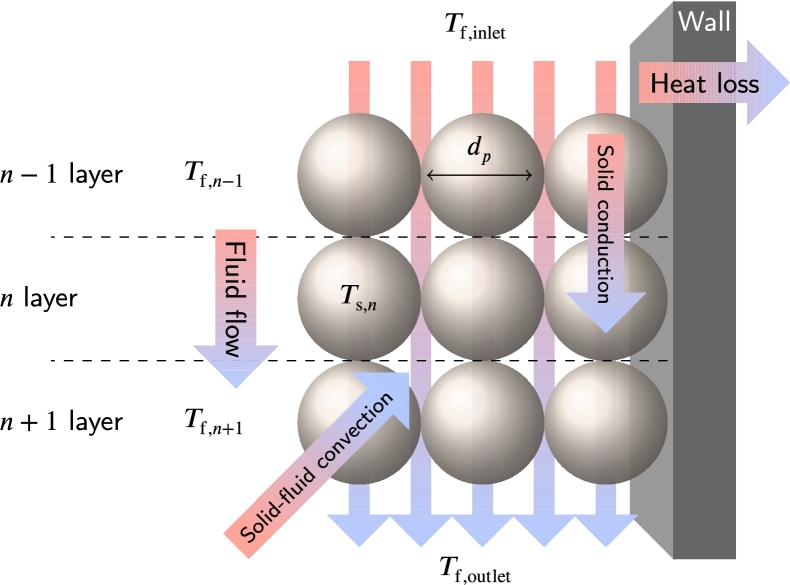
Schematic representation of a typical PB storage system based on the diagram presented in [16]. Notation is described in the text.

Packed-bed thermal storage involves the use of solids as a heat storage medium (bed) and a heat transfer fluid, HTF (gas or liquid), in direct contact with the solid (pieces or fragments of rocks, concrete, magnetite, etc.) with hollows or free space for HTF circulation in between. Both are contained in a single, thermally isolated tank (usually with a cylindrical symmetry) wherein a transition zone called the thermocline, characterized by a temperature gradient, separates hot and cold regions. This thermocline displaces in the axial direction during charge or discharge steps. Fig. 1 schematically represents the typical arrangement of a PB system. The fact that these systems use a single tank instead of the usual two-tank storage systems for solar applications contributes to their reduced cost.

It is customary, for convenience, to consider that during charge operation, the hot fluid goes into the deposit containing the solid from the top of the tank in order to improve thermal stratification because the hot region remains at the top and the cold at the bottom. During charging, several heat transfer mechanisms among the solid, HTF and tank walls change the temperatures of the three elements during the time and with the axial position. Refining models can also consider temperature gradients in the radial direction from the cylinder axis to the walls. During charge, the solid accumulates sensible energy associated with temperature variations or, if phase change materials (PCMs) are considered, also as latent heat energy [19]. At the time of discharge, a cold fluid is injected from the bottom of the tank that warms up with the previously stored thermal energy and leaves the deposit from the top at a higher temperature so it can be used through a thermodynamic cycle to be converted back into electric energy.

Heat transfer mechanisms during PB operation include conduction and radiation among all three elements, solid particles, fluid, and walls, and convection between the HTF and the particles and walls. As summarized by Calderón-Vásquez et al. [20] the basic qualitative parameters and variables for PB operation as storage systems include particle sizes, mass flow rate (relative to the section area), fluid and solid thermal properties, bed length, walls thermal isolation, void fraction (space in the deposit occupied by the HTF relative to the total volume), fluid inlet temperature and external temperature conditions.

### 1.2 Packed-bed applications in the renewable energy sector

#### 1.2.1 Concentrated solar power (CSP)

In concentrated solar power plants for power production, direct solar radiation is concentrated from a solar collector (parabolic trough, parabolic dish, central tower, etc.) towards a receiver to obtain high-temperature thermal energy. Afterwards, it is transformed into electric energy through a thermodynamic cycle (usually following Rankine or Brayton schemes) and an electric generator. A definitive advantage of CSP over other renewable technologies is its potential for hybridization (for instance, with other renewable systems as PV [21] and/or using a combustion chamber to guarantee a constant power output or to fit demand requirements [22]) and for coupling energy storage systems [23]. Because of the straightforward implementation of storage in CSP installations, they can be considered as storage systems.

Particularly in these plants, due to the day/night solar cycle and fluctuating weather conditions, TES is essential to reach commercial viability. Most standard TES systems applied to CSP rely on two-tank configurations for liquid storage where the hot and cold fluids are separated in different reservoirs. Investment and operational costs are relatively high, and the narrow range of operating temperatures for the usual storage fluids (molten salt is by far the most typical) between solidification and chemical decomposition has made necessary the research on different alternatives, such as solid or PB storage [24].

**Figure 2:**
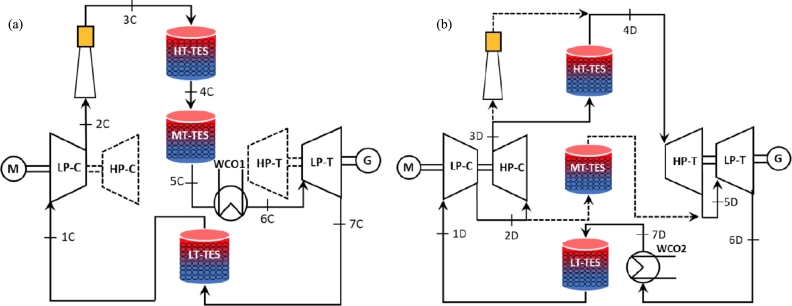
Scheme of the integration of PB-TES systems in a Brayton-based solar tower CSP plant [25]. a) Charging phase and b) discharging phase.

Fig. 2 depicts a possible configuration of a solar tower plant storing thermal energy through three PB containers and a multi-step closed Brayton cycle [25], [26]. The authors propose a feasible design for a 5 MW and 4 hours storage capacity giving a nominal exergetic roundtrip efficiency of about 60% at maximum temperatures slightly below 1000 K. They also conclude that plant components would frequently operate far from nominal conditions, and so, the development of specific models for off-design conditions together with suitable control strategies is significant for future evolution.

G. Alva et al. [27] have exhaustively investigated the possibilities of different materials (solid and liquid) for TES applications in solar energy systems. They compile the basic properties of an extensive list of materials, including costs per unit mass.

Baigorri et al. [19] have recently presented in a review the emerging concepts of TES storage for the new generation of CSP plants, including the possibility of PCM materials for storage and the estimated costs and technology readiness level (TRL) for different technologies. The authors stress the role that the joint action of CSP systems with storage and excess electricity storage systems (PTES, CAES, LAES, etc.) can play in the future design of renewable and dispatchable electric energy generation structure. A. El Fadar and O. Achkari present in [9] exhaustive tables with the operating CSP plants worldwide, detailing in each case, the type of TES system. The evolution of the levelized cost of electricity (LCoE) for these plants is analyzed, particularly its correlation with plants' storage capacity. Also, the possibilities of PCM materials on PB-TES are analyzed. Environmental issues related to CSP plants and TES are also summarized and commented on.

A. Gautam and R.P. Saini [28] studied the application of packed TES systems in CSP systems, including an extensive review of papers previous to 2020. Materials and design parameters related to heat transfer, pressure decay, and thermal losses are analyzed in detail. Khan et al. [15] compared sensible, latent, and thermo-chemical TES systems for solar applications, including describing real plants operating nowadays. The advantages and disadvantages of different HTF fluids and different technologies for the next generation of CSP plants were summarized.

Rahman et al. [3] focused on the economic aspects of different TES technologies, including life cycle assessment (LCA) aspects, and compiled a large amount of data for existing facilities. Trevisan et al. developed in [29] an economic model for a central tower CSP plant combined with a high-temperature packed-bed TES and performed a multi-objective optimization considering capital expenditure (CAPEX) and LCoS. M. Liu et al. [30] condensed in a review of 2016 TES systems for CSP applications from the point of view of the materials: HTF fluids and solids for beds, both for sensible and latent heat systems. Moreover, they analyzed the desirable properties of container materials depending on the storage medium. A. Palacios et al. [31] also analyzed different TES systems for CSP applications from the viewpoint of materials. The TRL of the main types of TES technologies was shown and analyzed with the corresponding strong points and scientific challenges. The number of patents and projects in each case was also quantified, distinguishing among CSP collector configurations. U. Pelay et al. concluded in [32] that in spite that most of the commercially operating CSP plants make use of sensible TES systems, the more significant energy density supported by latent and thermochemical TES ensures for them a bright foreground.

C. Suresh and R.P. Saini [33] compiled with great detail different materials for sensible and latent TES applications in CSP plants, giving tables with the most important physical and chemical variables determining their reliability for such applications. S. Trevisan et al. focused their work [34] on the option of using coatings to modify the effective properties of high-temperature PB thermal storage, essentially to achieve optimum values for thermal conductivities and emissivities looking for controlling charge process and thermocline degradation. Temperatures up to 1500 K were explored.

#### 1.2.2 Pumped thermal energy storage systems (PTES)

PTES, also known as pumped heat energy storage systems (PHES) (note that this acronym is used by several authors referring to pumped hydro energy storage; this is not the case in this paper), belong to those types of large-scale energy storage systems, specially suited for wind and PV applications that are under development nowadays. They are based on invertible heat pump cycles and are a particular case of a broader concept known as Carnot batteries [35]. Sharma and Mortazavi recently published an extensive review on the field [36].

**Figure 3:**
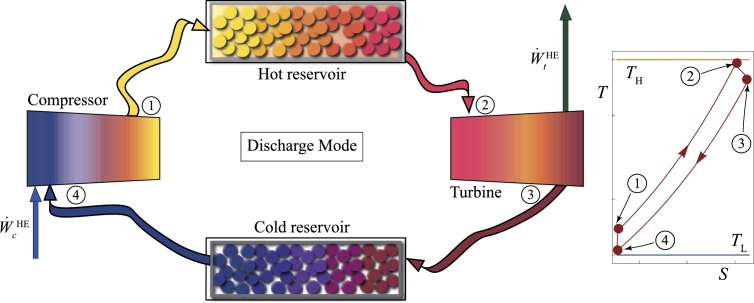
Schematic configuration of a PTES system following a Brayton cycle in the discharge process [38]. At the right, the corresponding *T* − *S* diagram is shown.

They are aimed to store energy during the hours with an excess of, for instance, wind or PV production. This aim is done through a heat pump (charge phase), which uses this electric energy to transfer heat from a cold to a hot reservoir. Thus, energy is stored as heat for the selected period (usually some hours). When electric energy is subsequently required, heat is transformed again in electricity through some thermodynamic cycle (discharge phase) working as a heat engine [8] usually following Rankine [37] or Brayton configurations [38]. Fig. 3 depicts an example of a Brayton PTES discharge cycle that uses PBs as thermal storage media [38]. It could also be interesting to directly transform the stored heat into useful heat, for instance, for industrial processes. Required storage periods would be not very long, from 4 to 10 hours. Delays of this order could be very significant in the future for smart grids [39].

Storage media could be liquid (different types of molten salts) or solid (bulk solids such as concrete or PB systems). A handicap for molten salts comes from the temperature operation interval (between solidification and decomposition), which is relatively narrow, approximately between 280 and 565 °C, which makes it compatible with CSP technology (see current working temperatures in Table 1). However, some mixtures such as KCl/MgCl_2_, MgCl_2_/NaCl/KCl, ZnCl_2_/NaCl/KCl, K_2_CO_3_/Na_2_CO_3_/Li_2_CO_3_, with degradation temperatures above 800 °C [40] also place molten salts in a region of interest for future applications in CSP as depicted in Table 1. Molten salt's actual electric storage capacity is about 75 kWh_*e*_/m^3^ [10] and sensible heat solid PB storage is predicted to reach values up to 233 kWh_*e*_/m^3^ [41].

A prototype PTES plant is located at Newcastle University, working at medium temperatures (about 500 °C) [42], rated at 150 kW_*e*_ and capable of storing 600 kWh_*e*_ of electricity with 8 hours storage. It has segmented PB reservoirs with magnetite pebbles. Recently published summary results [43] announce good round-trip efficiencies, among 55 and 76% at not too high costs. The authors estimated the costs of the prototype plant in a previous paper [44].

Research at fundamental and techno-economic levels is still needed to provide optimal plant configurations and adequate intervals for the most significant design parameters [2], [45]. Benato et al. include in [46] an estimation of the price per energy unit of a PTES system. For maximum temperatures in the range 550-1050 °C, a total storage volume of 300 m^3^, and different bed materials, they estimate prices between 50 and 180 euro/kWh. Zhao et al. [47] analyze different configurations for a Joule-Brayton PTES system and packed-bed storage (He as HTF and alumina). Apart from round-trip efficiencies (in the range of 64-66%), they estimate the total capital costs. The smallest value found for the latter among different plant configurations is 818 $/kWh.

Nowadays, research focuses on systems operating with Rankine or Brayton cycles and liquid or solid heat reservoirs. Brayton cycles can operate in a wide interval of temperatures and are expected to reach very high efficiencies at high temperatures. The working fluids in Brayton-like thermodynamic cycles for PTES are gases stable at high temperatures, chemically inert, cheap, and environment friendly [48]. These requirements limit the options to Ar and N_2_ (or air). Argon can reach higher temperatures for the same storage pressure ratios [35], although there is more experimental background in the turbo-machinery working with air.

Another line of research nowadays deals with the integration and operation of PTES systems with CSP plants [49], [50] or even simultaneously with PV, wind, and CSP energy sources [51]. In those configurations, PB storage plays a central role. Table 3 compares the most developed energy storage technologies at a large scale (pumped hydro storage) and several in-development techniques susceptible to incorporating PB thermal storage.

**Table 3:** Summary and comparison among the basic characteristics of several Electric Energy Storage (EES) systems, mechanical and thermo-mechanical. Own elaboration with data from [5], [52]. (a) Except for small plants. (b) Depends on the particular technology: TRL 5 for A-CAES (in-development) and 9 (pre-commercial) for other simpler arrangements.

EES	Mechanical	Thermo-mechanical		
	Pumped-hydro	PTES	CAES	LAES
Power rating	30-5000	10-150	0.5-1000	1-300
(MW)				
Energy capacity	100-20000	Up to GWh	0.1-2860	Up to GWh
(MWh)				
Energy density	0.5-2	10-100	0.5-20	50-200
(Wh/l)				
Energy efficiency	65-87	48-75	40-90	45-70
(%)				

thermal storage				

Response time	Mins.	s-Mins.	Mins.	Mins.
Discharge time	1-24 h	1-12 h	1-24 h	1-12 h
Storage duration	Hrs.-months	Hrs.-days	Hrs.-months	Hrs.-days

Site constraints	Yes	No	Yes${}^{\left(a\right)}$	No
TRL Level	9	2-5	5 -9	7-8
Maturity	Mature	In-develop.	(b)	In-develop.
Installed capacity	168 GW	-	431 MW	∼5 MW

#### 1.2.3 Compressed air energy storage (CAES)

The working process of the traditional CAES plants (so-called diabatic) is based on the use of one or several compressors in series that make use of electricity provided by wind or PV installations, for instance, in low-demand or high-production time spans to store compressed air in an underground huge reservoir, as abandoned mines, depleted gas fields, or rock or salt caverns. Before entering the next compressor or the reservoir, air must be cooled to reduce power consumption. This process generates large amounts of heat loss that diminishes system efficiency. When the compressed air is being discharged from the reservoir to be expanded in a turbine or a series of turbines, it is necessary to heat it to increase energy release efficiency. In diabatic CAES this is done through combustion chambers burning natural gas, generating greenhouse gases.

This concept was developed a long time ago, in the 1940s. However, there are at present two traditional CAES plants still in operation, mentioned in most reviews on the field, Huntorf in Germany (efficiency about 42%, operating from 1978) and McIntosh in the USA (about 54%) [53]. Among CAES advantages, its scalability (from micro-scale,<100 kW, to large scale, >50 MW), high lifetime, low self-discharge, and relatively low costs are worth-mentioning. On the contrary, its disadvantages include low round-trip efficiency and high response time. The scale of temperatures involved in different CAES researches ranges from low ones (< 200 °C) to medium temperatures (> 400 °C) [52].

Several alternative configurations have been proposed in the literature in order to improve efficiencies. These include, among others, adiabatic-CAES (A-CAES), advanced adiabatic-CAES (AA-CAES), and isothermal-CAES (I-CAES). An exhaustive classification can be found in [52]. It is expected that these configurations can have rated power from 0.1 to 1000 MW, storage capacity from 1 to 1000 MWh, round-trip efficiencies in the interval 75-95%, and a lower start-up time (less than a minute compared with 10 minutes for diabatic CAES) [8].

**Figure 4:**
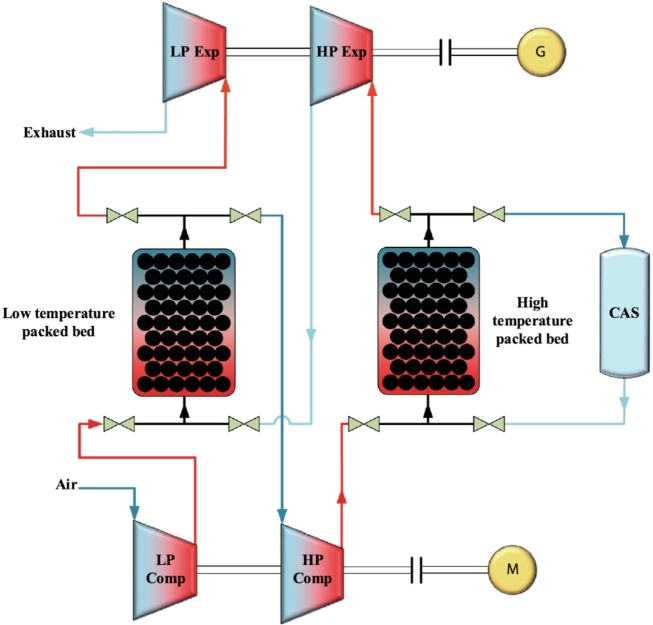
A-CAES scheme of a plant with two-stage compression and expansion, associated with two PB storages at low and high temperatures [53]. The compressed air reservoir is denoted as CAS.

In A-CAES the heat generated during the compression stage is afterward used to preheat air during expansion. However, it is still necessary fuel assistance to reach adequate turbine inlet temperatures. In AA-CAES, a TES system recovers the heat produced during the compression stage. Thus, during discharge, TES preheats the high-pressure air. It replaces the combustion chamber, increasing efficiency and making the system free of pollutant emissions [53]. Fig. 4 shows a possible scheme of integrating of PB TES in an AA-CAES installation. A demonstration plant called ADELE-ING (2013-17) was built with this concept [54]. Achieved efficiencies were about 60-70% with maximum temperatures around 600 °C at maximum pressures of 100 bar. In the low-pressure path, a PB TES was considered. As mentioned by Zhou et al. [53], one of the key directions for developing large-scale commercial CAES applications in the future passes through the research and optimization of TES systems in AA-CAES.

In isothermal-CAES (I-CAES) the idea is to approach ideally isothermal processes both in compression and expansion through particular designs of turbo-machinery, heat exchangers, or other techniques. Ideally, efficiencies of 100% could be reached, although the expected realistic ones would be around 80% [55].

Barbour et al. discuss in [56], [57] the TRL level of different CAES technologies based on in-development projects. They also include exciting comments on the components (compressors, expanders, heat exchangers, etc.) requirements for future successful technology deployment. In [52], Bazdar et al. present a feasibility study of incorporating CAES in energy systems focused on its combination with renewables, especially wind farms. They distinguish between small-scale and large-scale integration and compile abundant data. Related to costs, Cau et al. comment in [58] concerning CAES systems incorporating packed-bed storage that capital costs greatly depend on the particular technology, size, and site. Costs are also very sensitive to the operation strategy, that is, to the charging, storage, and discharging periods.

#### 1.2.4 Liquid air energy storage (LAES)

**Figure 5:**
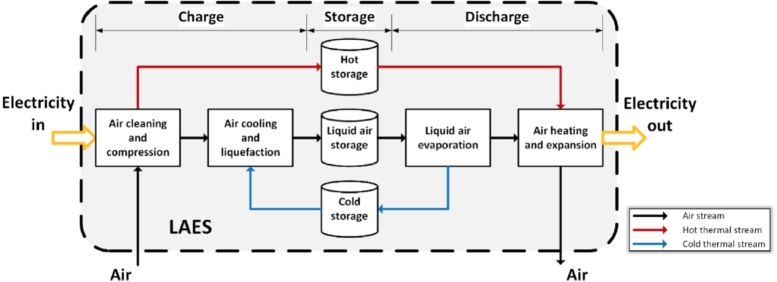
Charge and discharge processes in LAES [5].

Liquid air energy storage has some conceptual similitudes with CAES (and so, several authors consider it as a CAES subclass [52]), but also peculiarities that make it a particular thermo-mechanical technology for energy storage at large scale [5]. Similarly to other concepts, there are three basic processes in LAES, see Fig. 5. During charge, the air is taken from the ambient, purified, compressed using excess electricity, and cooled to get a gas-liquid phase transition. Once liquified, it is stored at almost ambient pressure in an ad-hoc reservoir at temperatures around -196 °C. Mostly, the thermodynamic cycles involved are Rankine-type. Apart from the waste heat from the liquefaction stage, other heat wastes or heat sources (as CSP in a hybrid configuration) can be utilized for improving the expansion process [11]. After the storage period, electricity can be produced by evaporating and expanding the working fluid in a set of turbines.

Probably, the most outstanding strong point of LAES against other technologies is its energy density (Vecchi et al. [5] outline that could be one or two orders of magnitudes over competitors), which implies reduced storage volumes, and the absence of site constraints for emplacement. LAES usually requires a TES subsystem that can be a PB vessel [59]. LAES is considered a close-to-maturity technology, although there are open challenges as its relatively low efficiency (40-50% nowadays without waste heat recuperation, 75-85% expected with recuperation), storage duration (shorter than CAES) and safety issues due to leakage (estimated as 0.05% by volume per day [60]). Table 3 outlines some of the basic parameters of LAES compared with other technologies. Olympios et al. [2] include in a review a unified thermo-economic comparison among several thermo-mechanical energy storage technologies, including LAES (see Figs. 30-35 therein).

A pilot scale plant was constructed by Highview Power Storage in Slough, United Kingdom, from 2008 to 2015 (350 kW/2.5 MWh) to get insights on the reliability of the technology [60]. A grid-scale plant developed by the same company in 2018 continued the development of this technology (5 MW/15 MWh), and there are projects to scale up to 50 MW/250 MWh [61].

### 1.3 Materials and HTFs

Different materials have been analyzed over the years to be used in packed-bed TES, both for the solid and the fluid phases. Due to their high heat capacity and low cost, silicon-based rock, magnetite, or sand are good candidates for the solid component clays. It is also possible to combine several of them simultaneously in a PB of mixed materials. There are two families of solid materials according to the way of storage thermal energy: sensible or latent.

#### 1.3.1 Sensible materials

Sensible materials exchange heat by contact (conduction or convection) and do not experience a phase transition. This group contains rocks, sand, clay, debris (demolition waste), alumina beads, etc. Their properties are practically constant with pressure and slightly vary with temperature. One of the most essential properties of sensible materials is the heat capacity. A high heat capacity is associated with a high energy density in the storage. However, many materials with high heat capacity also have a high thermal conductivity. High thermal conductivity is a problem because this reduces the energy stratification, *i.e.*, the thermocline. Therefore, the most appreciated materials are those with high heat capacity, low thermal conductivity, and low-cost price.

Table 4 summarizes the most usual materials. Detailed properties and extended tables can be found in [20], [62]. Concrete, castable ceramics, and inorganic industrial waste materials have been considered in several studies because of their relatively low cost. Materials costs data can be found in [20] (see Table 1 therein). Iron oxide and alumina can operate at high temperatures; over 600 °C and other aggregates have been analyzed for better thermophysical properties. Metal compounds such as cast iron or stainless steel have inherent benefits in thermophysical properties but have higher costs per kilogram. Reviews on sensible materials for PB applications can be found in [20], [62], [63]. One of the current research lines is using waste materials as rubble. However, these materials are too heterogeneous and have too variable properties to be tabulated. Its use is very interesting due to the low economic cost, but they require characterization before use [16].

**Table 4:** Thermodynamical data of main sensible thermal storage materials. Table extracted from [62] and [64].

Material	Heat capacity	Thermal conductivity
	⋅10^6^ [J/m^3^K]	[W/mK]
Aluminum oxide	3.2760	30.0 at 25 °C
Brick magnesia	3.3900	5.07
Cast iron	6.6123	29.3
Concrete	2.5310	0.9-1.3
Magnetite	3.811	−−
Pure iron	3.5694	73.0 at 20 °C

#### 1.3.2 Latent materials or phase change materials

Latent or phase change materials (PCM) present a state transition from solid to liquid in the working temperature range of the packed-bed. These materials have the advantage of storing a more significant amount of thermal energy due to the considerable amount of thermal energy necessary to carry out the phase change without increasing the temperature of the material. Due to this, PCM can store more thermal energy without using a higher working temperature than solid materials. On the other hand, PCMs are usually more expensive than solid ones. Some typical materials are NaNO_2_ composed materials.

When modeling materials that present a phase transition in the PB system, it is necessary to consider the existence of two well-defined states (solid and liquid) and the phase transition between them. Their respective heat capacities at constant pressure are well-known for solid and liquid states. When the temperature of the material reaches the temperature necessary for the transition, the phase change begins. At this point, it is required to know the latent fusion capacity of the material. Under ideal conditions, the transition is uniform and at a constant temperature, the reality is that the temperature is not uniform throughout the material, not even in the particles themselves, which may have a temperature gradient. A temperature range method is used to model the actual transition. With this method, two temperatures close to the melting temperature of the material ($T_{m1}$ and $T_{m2}$) are assumed. Below $T_{m1}$, it is considered solid and the capacity of the solid is applied. Above $T_{m2}$, it is assumed liquid, and the heat capacity of the liquid is used. In the interval between both, it is considered that there is a phase transition, and a heat capacity is applied to the material dependent on the latent heat of fusion (explicit usual equations for heat capacity, density, and conductivity of PCM can be found in A). However, if $c_{p}$ as a function of temperature is well known, it is possible to do an interpolation of $c_{p}$ in the temperature range.

In Pielichowska et al. [65], there is a list of potential candidates capable of being latent thermal energy storage materials. Those with high latent heat of fusion are the most valuable of all of them. For illustrative purposes, Table 5 shows some materials that can be highlighted, including inorganic compounds, sal hydrates, eutectic mixtures, organic paraffin, organic fatty acids, and organic sugars. In addition, it is crucial to choose a material with a melting temperature within the working range.

**Table 5:** Thermodynamical data of main latent thermal storage materials. For a more thorough list of materials see [65].

Material	Latent heat	Density	Melting point
	of fusion [J/g]	[kg/m^3^]	[°C]
KNO_3_	266	2.109 ⋅10^3^ (16 °C) [66]	333
NaNO_3_	172	2.257 ⋅10^3^ (solid [66])	307
KClO_4_	1253	2.52⋅10^3^[66]	527
LiH	2678	820 [66]	699
NaF	750	2.558⋅10^3^[66]	993
MgF_2_	936	3.15⋅10^3^[66]	1271
MgCl_2_⋅ 6 H_2_O	169	1.569⋅10^3^[67]	117
66.9% NaF +	−−	2940(solid 25 °C) [68], [69]	832
+33.1% MgF_2_			
Arachidic acid	227	824 [70]	74
n-tretadecane	229	762 [70]	6
Mannitol	341	1.49⋅10^3^[70]	165

Fadar et al. [9] have reviewed the desired properties for adequate PCM materials in PB applications and several experimental works in the field. Work lines concerning PCM materials (apart from reducing material costs) involve micro/macro encapsulation for enhancing heat transfer surface and the experimental research on thermal cycling looking for stability and chemical compatibility between PCM itself and surrounding materials [19].

#### 1.3.3 Heat transfer fluids

Heat transfer fluids for PBs have to be chemically and physically compatible with bed material. Moreover, they should have suitable properties such as specific heat, viscosity, thermal conductivity, and stability. They can be either liquid or gas. Air is the most used within gases because of its availability and insignificant cost, safety, and stability at high temperatures [71], [72]. Recently, steam and especially supercritical CO_2_, sCO_2_, are being investigated. The latter is in connection with high-temperature Brayton cycles because of its increased performance [19], [20].

Among liquids, thermal oils such as Therminol VP-1© and Therminol 66©, and molten salts are the most utilized. The highest operating temperature for thermal oils is about 400 °C. Molten salts could be used up to 550 °C and have relatively limited costs. Cascetta et al. [71] performed a direct comparative study considering the same bed (spherical particles of alumina) and three HTFs: air, oil, and molten salt. Other reviews and analyses can be consulted in [20], as well as costs per ton for the most usual HTFs (see Table 2 therein).

### 1.4 Previous studies

For completeness purposes and to show the progressive detail in the resolution of PB systems, some previous studies are presented in this section. A more extended explanation of the mathematical models is included in A.

In general, the energy conservation equations of a PB are complex to solve analytically. A series of simplifications on the initial equations can lead to analytical solutions. However, a more accurate description of the system requires numerical methods.

A series of simplifications can be used to solve the bed equations analytically. One approach considers all the magnitudes and properties of the fluid and the bed constant, independent of temperature and position. A second approach consists of neglecting the thermal conductivities in the bed equations. Among the resolutions found in the literature, it is possible to mention the following cases:

1. Riaz's analytical resolution [86], where a single-phase approximation is assumed. The bed and fluid temperatures are equal at each spatial and temporal instant, and the bed equation for a single-phase model can be solved analytically. In this approach, it is also assumed that the fluid heat capacity ($\rho \cdot c_{p}$) is negligible compared to that of the solid.
2. Zarty's analytical resolution [87], where a two-phase model is assumed, distinguishing bed temperature from fluid temperature. To simplify the equations and obtain an analytical resolution, Zarty assumes that the time derivative of the fluid is negligible compared to the rest of the terms. This approximation is only valid for fluids such as air.

The importance of numerical resolution compared to analytical can also arise from the variability of initial and boundary conditions of PB. They can be time or spatial-dependent and not constant, so this causes an impossible problem to solve through analytical solutions. In this sense,

3. Duffie's numerical resolution [88] proposes a *step-response* function to solve the analytical expressions for the fluid and solid temperatures of the Riaz analytical model.

Despite the long story of PB's, not many experimental setups have been built. In Table 6, compiled from [73], the main experimental setups with a size larger than 1 meter since 1986 are shown. Among them, there are only a few test rigs with high HTF inlet temperatures like Meier et al. [75]. Also, few are on a big scale, like Anderson et al. [78] or Ameen et al. [64], which has a big layered test rig.

**Table 6:** Main previous experimental studies of PB designs since 1986 with a length greater than 1 meter. Table from [73].

Studies	Year	Storage material	HTF	*T*_*max*_/*T*_*min*_	*L*/*D*_*int*_
				[°C]	[m]
Faas et al. [74]	1986	Granite rock and sand	Caloria © HT 43	295.5/179.2	12/18.2
Meier et al. [75]	1991	Magnesium silicate	Air	550/25	1.2/0.15
Pacheco et al. [76]	2002	Quartzite rock and sand	Molten salt	390/290	6/3
Yang et al. [77]	2012	Rock	Molten salt	500/300	2/1
Anderson et al. [78]	2014	Alumina	Air	120/20	10/9.56
Bruch et al. [79]	2014	Silica gravel and silica sand	Therminol 66 ©	≤300	3/1
Cascetta et al. [80]	2015/2016	Alumina beads	Air	300/25	1.8/0.58
Hoffmann et al. [81]	2016/2017	Quartzite rock	Rapeseed oil	210/160	1.8/0.4
Esence et al. [82]	2019	Cordieritic ceramic (plates)	Exhaust fumes	800/80	5/0.8
		and basalt (pebbles)	and air		
Keilany et al. [83]	2020	Alumina/Cofalit © rock	Jarysol © oil	300/100	2.64/1.2
Vannerem et al. [84]	2021	Alumina	Jarysol © oil	300/100	2.64/1.2
Xu et al. [85]	2022	Aluminum silicate	Water	≤60	3/1
Ameen et al. [64]	2023	Magnetite (86%) and silica (14%)	Argon	500/26	1.776/1.88

#### 1.4.1 Exergy analyses

Probably, the evaluation of performance in thermal energy storage systems is more extended by using the first law of thermodynamics (see Sec. 10), although this kind of evaluation has an explicit limitation: they do not show the energetic potential of the systems compared with their surroundings. A very interesting option is to do exergy analyses that explicitly involve thermodynamics' first and second laws. In a few words, exergy is the potential work that a thermodynamic system can do relative to a reference state, usually ambient conditions. Ideally, a thermal storage system should deliver energy at a temperature close to the inlet. Exergy recovery efficiency, which depends on the ratio of both temperatures, is a measure of efficiency in that sense [89]. It allows to identify and locating system losses, often more clearly than energetic performance.

Rosen presented in 2001 [90] a general theoretical scheme for analyzing exergy on stratified thermal energy storages. Li summarizes in [91] the most usual definitions of exergetic efficiencies for a charge, a discharge, and the overall process. In the same paper particular results are also condensed for PBs at different conditions, including sensible and latent heat systems.

Esence et al. [82] estimated the overall exergy efficiency of a 1.4 MW${}_{\text{th}}$ granular packed-bed system formed by basaltic rock and gas HTF. A hundred cycles with different mass flows were reproduced, and the overall exergetic efficiency reached an average value of around 85%. Temperatures reached 800 °C. González et al. [82] calculated the exergy delivered by a latent heat packed-bed for solar applications (parabolic trough plant with 20 and 50 MW_*e*_ with a storage capacity of about eight h) in particular locations including real meteorological conditions, with temperatures increasing between 100 and 275 °C. Very recently, also in the sector of solar applications, Calderón-Vásquez et al. [92] have performed exergy and cost analyses of four topologies of packed-bed at high temperatures operating with air. The analysis includes multi-objective Pareto calculations with TES costs per stored energy vs specific exergy losses.

There are in the recent literature other exergy calculations of systems including PBs for different applications, see for instance [4], [93], [94] in relationship with CAES applications and [10], [45] for PTES technologies.

### 1.5 Paper novelties, justification and objectives

As stated in this Introduction, PB thermal storage systems play a central role in developing technologies devoted to large-scale energy storage. Companies focused on the production and network integration of renewable energies are devoting substantial efforts to developing large-scale storage systems. This work will translate into a better accomplishment of electric energy demand at any time and better economic records, allowing increased investments in deploying of renewable power plants. Apart from the peculiarities of each of those technologies, PBs have several common ingredients. Its analysis has interesting physical and mathematical ingredients, such as theoretical modeling schemes, and methodologies.

Researchers from different fields use of PBs possibilities and try to integrate them into particular frameworks. However, theoretical modeling and the numerical methods required to obtain the aimed results are not trivial, and the literature has a great dispersion. This fact makes difficult to select the theoretical model that better matches a particular problem and to develop a numerical scheme for the obtention of the particular intended results. A great variety of types of solid materials and heat transfer fluids, temperature levels and the subsequent heat transfer mechanisms, and multiple physical effects (relevant or not for a particular problem) make it demanding for non-expert researchers to have a clear perspective of PB potentialities and analysis.

This paper aims to provide a comprehensive (instead of very extensive and technical) compilation of the different physical effects (and the corresponding hypotheses) that can be pertinent to a particular application. And concurrently, the options for numerically solving the considered model. There is a great variety of modeling schemes in the literature, sometimes with different languages, focused on very particular applications. Also, it is usual that the numerical methods dedicated to the computational solution of the problem are not explicitly or clearly explained. Thus, one of this paper's main novelties is to focus on a common ingredient of several large-scale energy storage systems, simultaneously condensing the main physical ingredients to be considered at different refinement models and developing numerical solution schemes for experts and non-experts.

The paper is organized as follows. The most relevant parameters and variables characterizing PB dynamics from a physical perspective are compiled in Sec. 2. A summary of representative and widely utilized models for describing the physics underneath these systems is presented in Sec. 3. Sections 4 and 5 summarize in an all-inclusive way reasonable numerical methods for solving the typical systems of partial differential equations arising from the physical models. Some practical and valuable tips are included. In Sec. 6, some guides for selecting the most appropriate physical model in a particular application and the corresponding solving method are exposed from the perspective of some particular cases. The influence of spatial and temporal steps in computational solving methods is discussed in Sec. 7. Section 8 presents several comparisons among some relevant experimental results (liquid and gas HTFs, sensible heat transfers or PCMs, different temperature levels, etc.) and the corresponding simulation predictions. Section 9 is a brief one devoted to calculating of pressure drops in the case of gaseous HTFs. Finally, before the conclusions, some ways to measure the energy stored in these thermal storage systems and their efficiency are presented. Also, some particular numerical cases are studied and commented on. These are done in Secs. 10 and 11. Detailed tables with the experimental setups considered for validations and detailed equations employed in the resolution of PB dynamics are compiled in some Appendices.

## 2 Dynamical properties of packed-bed storage systems

To fix a notation, it will be considered that the reference packed-bed system is made up of a storage tank of length *L*, internal diameter $D_{int}$, one or several layers of insulation with their respective thickness until reaching the final external diameter of the bed $D_{ext}$, which external surface is in contact with air at room temperature $T_{\infty }$. If the geometric shape of the bed is cylindrical, the cross-section is known $A_{t}=\pi {\left(D_{int}/2\right)}^{2}$. This geometric configuration will be assumed in this study.

The dynamical equations that describe the energy transport inside the tank consist, in a general form, of two coupled equations describing the contribution to energy transfers between the two phases (liquid and solid) inside the tank and the heat transfer with the surroundings, escaping through the walls of the container. The level of detail in the mathematical description and the approach to solving the problem can differ, as mentioned in the Introduction and as will be addressed later in this work. Nonetheless, the most common design and dynamical parameters involved in all these models are described below.

Void fraction (*ϵ*) defines the amount of empty space within the bed. It is the ratio between the volume not occupied by the storage material and the total volume of the PB.

(1) $$\epsilon =\frac{V_{HTF}}{V_{total}}=1-\frac{V_{solid}}{V_{total}}.$$

The following assumptions will be made: the particles that make up the bed are uniform, spherical, homogeneous, and have a diameter $d_{p}$ (. 1).

The parameter $a_{s}$ represents the surface area (*A*) of the particles per unit volume, using Eq. (1) [16]:

$$a_{s}=\frac{A_{solid}}{V_{total}}=\frac{6(1-\epsilon )}{d_{p}}.$$

The parameter $a_{b}$ represents the ratio between the internal area of the bed and its total volume, through which the heat leaks to the outside. A cylindrical bed and negligible leaks from the top and bottom covers are supposed, thus:

$$a_{b}=\frac{A_{int}}{V_{total}}=\frac{2}{r_{in}}.$$

### 2.1 Fluid velocity

The fluid velocity is a determining parameter when modeling the heat transfer in the bed. The mass flow rate is defined as the amount of mass per unit of time that enters the bed or crosses any cross-section. By conservation of mass, this magnitude must be constant. However, the volumetric flux (*Q*) and superficial velocity ($U_{s}$) will be temperature-dependent:

$$Q=\frac{\overset{˙}{m}}{\rho_{f}},U_{s}=\frac{Q}{A_{t}}.$$

Also, the interstitial velocity can be defined as follows [16],

$$u=\frac{U_{s}}{\epsilon }=\frac{\overset{˙}{m}}{\epsilon \rho_{f}A_{t}}.$$

When considered a real fluid, the heat transfer fluid will have properties dependent on temperature and pressure. It is possible to obtain a series of values depending on temperature and pressure in the range between the working values. With these values, it is usual to perform linear regressions for the following parameters: thermal conductivity ($K_{f}$), heat capacity at constant pressure ($c_{p,f}$), density ($\rho_{f}$), and dynamic viscosity of the fluid ($\mu_{f}$). This approach allows us to obtain the kinematic viscosity of the fluid, *ν*, and Prandtl number, *Pr* [16].

$$\nu_{f}=\mu_{f}/\rho_{f},Pr=\frac{\mu_{f}\cdot c_{p,f}}{K_{f}}.$$

Prandtl number represents the ratio between kinematic viscosity and thermal diffusivity. Bed density ($\rho_{s}$), heat capacity ($c_{p,s}$), and its conductivity ($K_{s}$) will vary depending on the chosen material and will be temperature dependent but not pressure dependent.

### 2.2 Fluid-bed convection coefficient

The convection heat transfer coefficient between the fluid and the bed is the most determining parameter of all those which govern the thermal behavior of the bed. This coefficient quantifies the direct heat transfer between the fluid and the bed. It depends on the particle Reynolds number, $R_{ep}$, and the Nusselt number, *Nu*. Reynolds number depends on the packing material diameter, $d_{p}$, and it is defined as [16],

$$R_{ep}=\frac{\epsilon \cdot u\cdot d_{p}}{\nu_{f}}.$$

Different correlations can be found for the Nusselt number which depend on the specific system properties. An appropriate correlation for fluid-rock heat transfer considering a single-size sphere when $10\leq R_{ep}\leq 10^{4}$ is given by [95]

$$N_{u}=2.0+1.1\cdot R_{ep}^{0.6}\cdot Pr^{1/3},$$

leading to the final expression of the fluid-rock convection coefficient [16]:

$$h=\frac{K_{f}\cdot N_{u}}{d_{p}}.$$

### 2.3 Fluid-pipe convection coefficient

The heat convection coefficient between the fluid and the wall surrounding the packed-bed (pipe) depends on a new Nusselt number for forced convection [96], [97], [98].

$$N_{u,in}=2.57R_{ep}^{1/3}Pr^{1/3}+0.0936R_{ep}^{0.8}Pr^{0.4},$$

and the fluid-pipe convection coefficient for forced convection is [97]:

$$h_{in}=\frac{K_{f}\cdot N_{u,in}}{d_{p}}.$$

### 2.4 Insulation-outdoor air convection coefficient

The external insulation of the PB is surrounded by air, therefore there is a free convection process. The convection coefficient between the external air and the insulation is determined by the following parameters:

Grashof number [99]:

$$G_{r}=\frac{\frac{g}{T_{\infty }}\left(T_{wall,ext}-T_{\infty }\right)\cdot L^{3}}{\nu_{air}^{2}},$$

Rayleigh number [99]:

$$R_{a}=G_{r}\cdot Pr_{air},$$

and the Nusselt number considering a laminar flow ($10^{5}<R_{a}<10^{9}$) [100]:

$$N_{u,ext}=0.68+\frac{0.67R_{a}^{1/4}}{{\left(1+{\left(0.492/Pr_{air}\right)}^{9/16}\right)}^{4/9}},$$

leading to the final expression for the external convection coefficient for vertical free convection cylinders [101]:

$$h_{ext}=\frac{K_{f}\cdot N_{u,ext}}{L}.$$

### 2.5 Thermal conductivities

From the thermal conductivity of the fluid and the bed ($K_{f}$ and $K_{s}$, respectively), the effective conductivities are obtained according to the direction of heat propagation: radial or axial. Radial and axial conductivities will be used for symmetry since the problem has cylindrical geometry, so it will be a one-dimensional (1D) problem if the radial variation of temperature is neglected or two-dimensional (2D) model approach if the radial variation of temperature is considered. In addition, depending on the selected model to describe the PB, a total conductivity will be used for both fluid and bed combined homogeneous phases or two different conductivities for the different heterogeneous phases. In the one-phase homogeneous model, effective thermal conductivities account for conduction and convection (ignoring radiation) between both phases in the axial and radial directions. These are given by [97], [102],

$$K_{t,eff,z}=K_{f}\cdot \left(\frac{K_{e}^{0}}{K_{f}}+0.5Pr\cdot R_{ep}\right),$$

for the total axial conductivity, where $K_{e}^{0}$ is the stagnation effective thermal conductivity calculated from

$$K_{e}^{0}=K_{f}{\left(\frac{K_{s}}{K_{f}}\right)}^{m};m=0.28-0.757\log(\epsilon )-0.057\log\left(\frac{K_{s}}{K_{f}}\right),$$

and

$$K_{t,eff,r}=K_{f}\left(\frac{K_{e}^{0}}{K_{f}}+0.1Pr\cdot R_{ep}\right)$$

accounts for the total effective radial conductivity.

For two-phase heterogeneous models, fluid and bed thermal conductivities can be calculated separately as follows [97], [102].

For the fluid:

• Axial and radial effective thermal conductivity
  $$K_{f,eff,z}=K_{f,eff,r}=0.7\epsilon K_{f};\;if\;R_{ep}\leq 0.8,K_{f,eff,z}=K_{f,eff,r}=0.5PrR_{ep}K_{f};\;if\;R_{ep}>0.8.$$

For bed storage material:

• Axial effective thermal conductivity
  $$K_{\text{s,eff,z}}=K_{t,eff,z}-K_{f,eff,z}.$$
• Radial effective thermal conductivity
  $$K_{s,eff,r}=K_{t,eff,r}+K_{f,eff,r}.$$

### 2.6 Effective heat loss coefficient

The heat transfer coefficient *U* is a parameter that determines the total effective losses due to heat leakage through the bed wall and to the outside. These losses are made up of thermal losses due to conduction through the wall and the insulation, losses due to convection between the working fluid and the wall, and convection between the external insulation and the air. Losses through thermal radiation can also be included when the bed temperature is very high. As described by Lienhard [103], the overall heat loss coefficient corresponds to an equivalent thermal resistance ($R_{equiv}$) per unit heat transfer area (*A*).

$$U\cdot A=\frac{1}{R_{equiv}}=\frac{1}{\sum \frac{1}{R_{i}}}.$$

**Figure 6:**
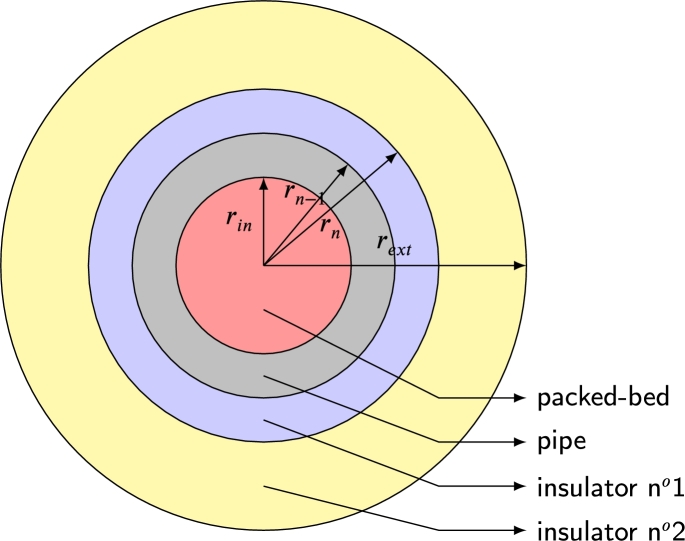
Cross-section of the storage system. The thermal properties of the different layers are included in the analysis of the heat losses between the storage tank and the environment at ambient temperature (*T*_∞_).

**Figure 7:**
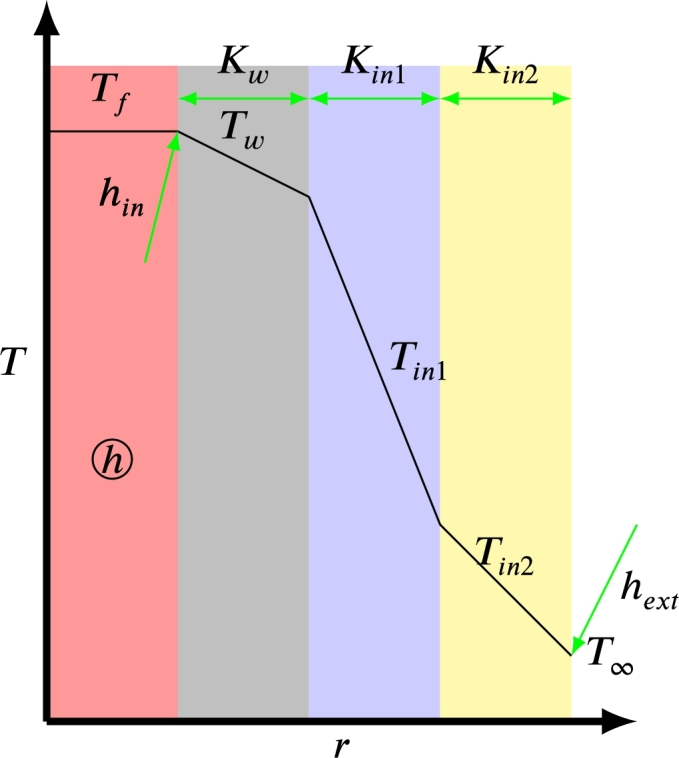
Losses through the pipe and insulator walls. Temperature will decrease near to the outside of PB.

For the case discussed, a PB with cylindrical geometry, the parameter *U* is defined in the following way [104],

$$U^{-1}=r_{in}\cdot \left(\frac{1}{r_{in}h_{in}}+\sum_{n=1}^{N}\frac{\log\left(r_{n}/r_{n-1}\right)}{K_{n}}+\frac{1}{r_{ext}h_{ext}}\right),$$

where *N* is the total amount of materials between storage material and external air (including pipe and insulators). The parameter $r_{in}$ is the internal radius of the bed and, $r_{ext}$ is the external radius of the last layer of the previous insulating material of the bed, which is in direct contact with external air. The layers of pipe and intermediate insulators correspond to radius $r_{n}$, and each has a thickness of $r_{n}-r_{n-1}$. Fig. 6 contains a schematic representation of the different layers in the particular case of two insulation surfaces. Fig. 7 shows the temperature variation through packed-bed walls and insulators.

For high bed temperatures, the radiation losses from the Stefan-Boltzmann law cannot be neglected:

$$h_{rad}=\frac{\varepsilon_{mat}\sigma \left(T^{4}-T_{\infty }^{4}\right)}{T-T_{\infty }},$$

where $0\leq \varepsilon_{mat}\leq 1$ is the emissivity of the material and $\sigma =5.67\cdot 10^{-8}\;(W/m^{2}K)$ is the Stefan-Boltzmann constant. The temperature *T* refers to the outside temperature of the insulator. Therefore, the effective loss coefficient is as follows:

$$U^{-1}=r_{in}\cdot \left(\frac{1}{r_{in}h_{in}}+\sum_{n=1}^{N}\frac{\log\left(r_{n}/r_{n-1}\right)}{K_{n}}+\frac{1}{r_{ext}\left(h_{ext}+h_{rad}\right)}\right).$$

## 3 Time-dependent solution to the heat equation

This section states and analyzes the energy conservation equations for a PB system under different physical assumptions. From the dynamic parameters described above, the temperature field inside the storage tank can be obtained and compared using different approaches depending on the intended application, the required precision level, and the computational resources.

According to the cylindrical geometry of a bed, there is rotational symmetry in the axial direction; therefore, the three dimensions can be reduced to two dimensions (2D): in the axial and radial directions. Simplifying further, by assuming that the relevant dimension of the system is its length, the radial dimension can be suppressed and can be assumed only an axial dependence on the temperature in the bed (1D). Next, some usual models in the literature are stated [105].

### 3.1 One-phase models

One or single-phase models consist of approximating the solid phase and the fluid by a single set. The temperature of the bed and the fluid are assumed to be equal at each point instantaneously. In this way, both equations are reduced to a single one, and there is only one equivalent temperature, equal to that of the bed and the fluid simultaneously ($T_{f}=T_{s}=T$). These models can be applied when the bed's thermal conductivity and heat capacity are very high compared to those of the fluid, for example, rock and air [97]. The energy conservation equation of the single-phase model in one dimension (1 phase - 1D) can be written as follows:

$$\left[(1-\epsilon )\rho_{s}c_{p,s}+\epsilon \rho_{f}c_{p,f}\right]\frac{\partial T}{\partial t}+\rho_{f}c_{p,f}\epsilon u\frac{\partial T}{\partial z}=K_{t,eff,z}\frac{\partial^{2}T}{\partial z^{2}}+Ua_{b}\left(T_{\infty }-T\right),$$

and the energy equation of single phase model in two dimensions (1 phase - 2D) can be read as:

$$\left[(1-\epsilon )\rho_{s}c_{p,s}+\epsilon \rho_{f}c_{p,f}\right]\frac{\partial T}{\partial t}+\rho_{f}c_{p,f}\epsilon u\frac{\partial T}{\partial z}=K_{t,eff,z}\frac{\partial^{2}T}{\partial z^{2}}+K_{t,eff,r}\left[\frac{\partial^{2}T}{\partial r^{2}}+\frac{1}{r}\frac{\partial T}{\partial r}\right].$$

### 3.2 Two-phase models

In these models, the temperature of the solid and the fluid are treated separately. They are more realistic models that assume a temperature difference between rock ($T_{s}$) and HTF ($T_{f}$). Their range of applicability in particular cases is greater than that of the one-phase model, as it will be commented on later.

#### 3.2.1 Schumann's model

It is a simple model that describes the bed, neglecting the thermal conductivity of the fluid and the rock. Furthermore, as a simplistic model, the heat leaks from the bed to the outside environment are neglected [17]. Energy equations of Schumann's model in one dimension (2 phases - 1D) are defined in the following way:

$$\epsilon \rho_{f}c_{p,f}\overset{\text{enthalpy}}{\overset{︷}{(\frac{\partial T_{f}}{\partial t}}}+\overset{\text{convection}}{\overset{︷}{u\frac{\partial T_{f}}{\partial z})}}=\overset{\text{convection}}{\overset{︷}{ha_{s}\left(T_{s}-T_{f}\right)}},\overset{\text{enthalpy}}{\overset{︷}{(1-\epsilon )\rho_{s}c_{p,s}\left(\frac{\partial T_{s}}{\partial t}\right)}}=\overset{\text{convection}}{\overset{︷}{ha_{s}\left(T_{f}-T_{s}\right)}}.$$

Note that there is no possibility of rewriting this model in two dimensions, due to the absence of axial or radial thermal conductivities.

#### 3.2.2 Continuous solid phase model

This model is an extension of the Schumann model. In this case, the thermal conductivity of the materials and HTF involved is taken into account. Heat leaks are included through the effective loss coefficient (*U*), which provides heat conductivity, convection, and radiation losses. The equations of the continuous solid phase model in one dimension (2 phases - 1D) are:

$$\epsilon \rho_{f}c_{p,f}\overset{\text{enthalpy}}{\overset{︷}{(\frac{\partial T_{f}}{\partial t}}}+\overset{\text{convection}}{\overset{︷}{u\frac{\partial T_{f}}{\partial z})}}=\overset{\text{conduction}}{\overset{︷}{\frac{\partial }{\partial z}\left(K_{f,eff,z}\frac{\partial T_{f}}{\partial z}\right)}}+\overset{\text{convection}}{\overset{︷}{ha_{s}\left(T_{s}-T_{f}\right)}}+\overset{\text{heat leaks}}{\overset{︷}{Ua_{b}\left(T_{\infty }-T_{f}\right)}},$$

$$\overset{\text{enthalpy}}{\overset{︷}{(1-\epsilon )\rho_{s}c_{p,s}\frac{\partial T_{s}}{\partial t}}}=\overset{\text{conduction}}{\overset{︷}{\frac{\partial }{\partial z}\left(K_{\text{s,eff,z}}\frac{\partial T_{s}}{\partial z}\right)}}+\overset{\text{convection}}{\overset{︷}{ha_{s}\left(T_{f}-T_{s}\right)}}$$

The equations of the continuous solid phase model in two dimensions (2 phases - 2D) can be written through the thermal gradient in a radial direction:

$$\epsilon \rho_{f}c_{p,f}\left(\frac{\partial T_{f}}{\partial t}+u\frac{\partial T_{f}}{\partial z}\right)=\frac{\partial }{\partial z}\left(K_{f,eff,z}\frac{\partial T_{f}}{\partial z}\right)+K_{f,eff,r}\left[\frac{\partial^{2}T_{f}}{\partial r^{2}}+\frac{1}{r}\frac{\partial T_{f}}{\partial r}\right]+ha_{s}\left(T_{s}-T_{f}\right),\left(1-\epsilon \right)\rho_{s}c_{p,s}\frac{\partial T_{s}}{\partial t}=\frac{\partial }{\partial z}\left(K_{\text{s,eff,z}}\frac{\partial T_{s}}{\partial z}\right)+K_{s,eff,r}\left[\frac{\partial^{2}T_{s}}{\partial r^{2}}+\frac{1}{r}\frac{\partial T_{s}}{\partial r}\right]+ha_{s}\left(T_{f}-T_{s}\right).$$

#### 3.2.3 Thermal gradient model in solid particles

In this model, a thermal gradient inside the particles of the packed-bed is considered. Thus, finite (non-punctual) size particles are modeled. Furthermore, the temperature in the particles changes due to contact with the fluid on their surface (convection) and its internal conduction through the interior of the particles. Another assumption is that there is no heat conduction between bed particles. In this way, the second rock equation in two-phase models equations is replaced by the equation that describes the temperature gradient in the particle. The equations of the thermal gradient model in solid particles in one dimension (2 phases - 1D) are:

$$\epsilon \rho_{f}c_{p,f}\left(\frac{\partial T_{f}}{\partial t}+u\frac{\partial T_{f}}{\partial z}\right)=\frac{\partial }{\partial z}\left(K_{f,eff,z}\frac{\partial T_{f}}{\partial z}\right)+ha_{s}\left(T_{s}{|}_{\eta =d_{p}/2}-T_{f}\right)+Ua_{b}\left(T_{\infty }-T_{f}\right),\rho_{s}c_{p,s}\frac{\partial T_{s}}{\partial t}=K_{s}\left[\frac{\partial^{2}T_{s}}{\partial \eta^{2}}+\frac{2}{\eta }\frac{\partial T_{s}}{\partial \eta }\right],$$

where *η* is the radial component of particles. Also, the thermal gradient model can be written in two dimensions (2 phases - 2D), using again the temperature gradient in radial direction through the bed:

$$\epsilon \rho_{f}c_{p,f}\left(\frac{\partial T_{f}}{\partial t}+u\frac{\partial T_{f}}{\partial z}\right)=\frac{\partial }{\partial z}\left(K_{f,eff,z}\frac{\partial T_{f}}{\partial z}\right)+ha_{s}\left(T_{s}{|}_{\eta =d_{p}/2}-T_{f}\right)+K_{f,eff,r}\left[\frac{\partial^{2}T_{f}}{\partial r^{2}}+\frac{1}{r}\frac{\partial T_{f}}{\partial r}\right],\rho_{s}c_{p,s}\frac{\partial T_{s}}{\partial t}=K_{s}\left[\frac{\partial^{2}T_{s}}{\partial \eta^{2}}+\frac{2}{\eta }\frac{\partial T_{s}}{\partial \eta }\right].$$

### 3.3 Three-phase models

Up to here, the energy equations for the two phases are set for HTF and storage material. In addition to the fluid and bed, more materials can be involved in the TES, as the metal of the container wall. Three-phase models include the bed wall in the simulations for more realistic modeling. Equations of the three-phase model with wall contribution in one dimension (3 phases - 1D) can be written as follows:

$$\epsilon \rho_{f}c_{p,f}\left(\frac{\partial T_{f}}{\partial t}+u\frac{\partial T_{f}}{\partial z}\right)=\frac{\partial }{\partial z}\left(K_{f,eff,z}\frac{\partial T_{f}}{\partial z}\right)+ha_{s}\left(T_{s}-T_{f}\right)+h_{int}a_{b}\left(T_{w}-T_{f}\right),\left(1-\epsilon \right)\rho_{s}c_{p,s}\frac{\partial T_{s}}{\partial t}=\frac{\partial }{\partial z}\left(K_{\text{s,eff,z}}\frac{\partial T_{s}}{\partial z}\right)+ha_{s}\left(T_{f}-T_{s}\right),x_{w}\rho_{w}c_{p,w}\left(\frac{\partial T_{w}}{\partial t}\right)=\frac{\partial }{\partial z}\left(K_{w}x_{w}\frac{\partial T_{w}}{\partial z}\right)+x_{w}h_{int}a_{b}\left(T_{f}-T_{w}\right)+x_{w}h_{ext}a_{b,ext}\left(T_{\infty }-T_{w}\right).$$

However, the system with an additional equation is more difficult to solve than only with one or two. To include wall effects without needing a third equation, an effective bed density can be used, where wall effects are included. The effective density can be written using the mass of tank walls ($m_{w}$), the walls heat capacity ($c_{p,w}$), and the volume of tank walls ($V_{b}$) [79]:

(2) $$\rho_{s,eff}=\rho_{s}+\frac{m_{w}c_{p,w}}{\left(1-\epsilon \right)c_{p,s}V_{b}},$$

thus, the energy equations of a two-phase model (continuous solid phase model) in one dimension considering the bed wall corrections (2 phases - 1D) can be written as:

$$\epsilon \rho_{f}c_{p,f}\left(\frac{\partial T_{f}}{\partial t}+u\frac{\partial T_{f}}{\partial z}\right)=\frac{\partial }{\partial z}\left(K_{f,eff,z}\frac{\partial T_{f}}{\partial z}\right)+ha_{s}\left(T_{s}-T_{f}\right)+Ua_{b}\left(T_{\infty }-T_{f}\right),\left(1-\epsilon \right)\rho_{s,eff}c_{p,s}\frac{\partial T_{s}}{\partial t}=\frac{\partial }{\partial z}\left(K_{\text{s,eff,z}}\frac{\partial T_{s}}{\partial z}\right)+ha_{s}\left(T_{f}-T_{s}\right).$$

### 3.4 Other refinements

Other analyses and approaches to particular problems that can arise in specific situations can be found in the literature. Two especially interesting problems are next mentioned: multisized particles in the bed and the importance of radiation heat exchange at high temperatures.

Authors such as Odenthal et al. [106] have proposed the extension of Schumann's model for two types of particles in the bed (bimodal) with different sizes. Two solid-phase equations are considered, and another for the HTF, so a three-equation model arises. Convective heat transfer and surface area of particles coefficients are doubled, one for each species in the bed. Even more refined is the mixed approach developed by Esence et al. [107] where both species in the bed are considered to have quite different sizes, so a dispersed concentric model is assumed for the largest and a single-phase model is taken for the smallest. The model also explicitly considers the energy balance for the walls, and in consequence, four equations are to be solved (energy balances for the wall, small and large particles, and a continuity equation for the fluid velocity).

Radiation heat exchange is essential at high temperatures, probably over 900 °C. The direct consideration of radiation requires the calculation of view factors among the elements of the storage system, but this has a prohibitive computational cost. Thus, the most usual is considering effective thermal conductivities, fitted from experimental results [72], [20]. Other alternatives as short- or long-range models and the efficient calculation of view factors, can be seen in [20].

### 3.5 Initial and boundary conditions

#### 3.5.1 Initial conditions

The initial temperature of both phases can be determined from experimental data as done by Cascetta et al. [80] or by modeling consecutive charge/discharge cycles. In this work, given the solid phase and the air as HTF, the initial temperature will be equal to the ambient temperature to begin loading the bed, Eq. (3),

(3) $$T_{s}\left(t=0\right)=T_{f}\left(t=0\right)=T_{\infty }$$

and to begin unloading the bed, it will be the final temperature upon completion of charging.

#### 3.5.2 Boundary conditions

Boundary conditions are also necessary to solve the differential equations. They are a series of additional equations necessary to solve the temperatures at the boundaries of the problem domain, that is, at the lower and upper edges of the bed. Neumann boundary conditions are applied, establishing a condition written as a partial derivative on the domain boundary. The condition is zero heat leakage at the domain edge. In addition, there is a constant fluid inlet temperature over time to carry out the charge. The general boundary conditions in one-dimension models are the following:

$$T_{f}\left(z=0\right)=T_{f,inlet},{\frac{\partial T_{f}}{\partial z}|}_{z=L}=0,{\frac{\partial T_{s}}{\partial z}|}_{z=0}={\frac{\partial T_{s}}{\partial z}|}_{z=L}=0.$$

Furthermore, in two dimensions models, the following additional boundary conditions are needed:

$${\frac{\partial T_{f}}{\partial r}|}_{r=0}={\frac{\partial T_{s}}{\partial r}|}_{r=0}=0,{\frac{\partial T_{s}}{\partial r}|}_{r=D_{in}/2}=0,{-K_{f,eff,r}\frac{\partial T_{f}}{\partial r}|}_{r=D_{in}/2}=U{\left(T_{\infty }-T_{f}\right|}_{r=D_{in}/2}).$$

In the special case of thermal gradient in solid particles model, a pair of additional boundary conditions are needed:

$${\frac{\partial T_{s}}{\partial \eta }|}_{\eta =0}=0,{K_{s}\frac{\partial T_{s}}{\partial \eta }|}_{\eta =d_{p}/2}=h{\left(T_{f}-T_{s}\right|}_{\eta =d_{p}/2}).$$

## 4 Approach to finite elements and meshing

The equations proposed in the models are coupled differential equations. The simplest ones can be solved analytically, but most will require a numerical method.

**Figure 8:**
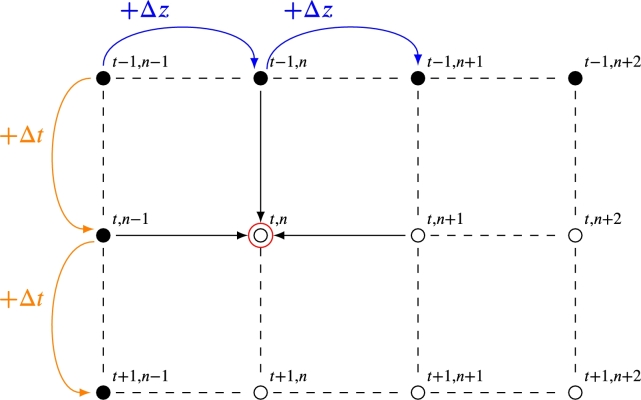
Temporal and spatial mesh of a PB. Spatial discretization of a constant time in each row. Black dots have well-known temperatures. White dots have unknown temperatures.

The finite element method establishes a spatial and temporal mesh to discretize the equations based on the independent variables (space and time). In the simplest case, for only one axial spatial dimension, the length of the bed is divided into $N_{z}$ equidistant spatial nodes of length Δ*z*, that is, $N_{z}=L/\Delta z+1$. The space between nodes will be the minimum spatial division of the bed; see Fig. 8. For the temporal dimension, a discretization will be done again. The total or maximum charge time ($t_{max}$) will be divided into a total of $N_{t}$ temporary nodes, with an interval of Δ*t* between them, so $N_{t}=t_{max}/\Delta t+1$. This allows the creation of a mesh, with the nodes at every specific temporal and spatial interval. The next step consists of solving the model's differential equations numerically, obtaining the fluid and bed temperatures in each spatial node for a constant time. Once this step is completed, the algorithm is repeated but in the spatial nodes, corresponding to the following time interval. In this iterative way, the temperatures are calculated numerically for the spatial nodes through the PB length and for all time steps until the charging time ends.

### 4.1 Euler method

The Euler method is a first-order finite difference method (FDM). It consists of approximating the partial derivatives by increments of the function's value. Next, two variants of the Euler method are explained for calculating temperature evolution in a packed-bed: the explicit (forward) and the implicit (backward). The variables dependent on temperature are evaluated with the immediately previous values of temperatures.

#### 4.1.1 Explicit Euler method

The first variant of the Euler method is the explicit one. It consists of approximating the partial derivatives by forward increments of the function. The spatial and temporal increases of derivatives result as follows:

(4) $$T\equiv T_{n}^{t},$$

(5) $$\frac{\partial T}{\partial t}=\frac{T_{n}^{t}-T_{n}^{t-1}}{\Delta t},$$

(6) $$\frac{\partial T}{\partial z}=\frac{T_{n}^{t}-T_{n-1}^{t}}{\Delta z},$$

(7) $$\frac{\partial^{2}T}{\partial z^{2}}=\frac{T_{n+1}^{t-1}-2T_{n}^{t-1}+T_{n-1}^{t-1}}{\Delta z^{2}}.$$

Using Eqs. (4)-(7), the one-phase model can be rewritten in one dimension (one-phase -1D), in terms of finite elements:

$$\left[\left(1-\epsilon \right)\rho_{s}c_{p,s}+\epsilon \rho_{f}c_{p,f}\right]\frac{T_{n}^{t}-T_{n}^{t-1}}{\Delta t}+\rho_{f}c_{p,f}\epsilon u\frac{T_{n}^{t}-T_{n-1}^{t}}{\Delta z}=K_{t,eff,z}\frac{T_{n+1}^{t-1}-2T_{n}^{t-1}+T_{n-1}^{t-1}}{\Delta z^{2}}+Ua_{b}\left(T_{\infty }-T_{n}^{t}\right).$$

Also, Schumann's model in one dimension (tow-phase- 1D) can be rewritten using the explicit Euler method:

$$\epsilon \rho_{f}c_{p,f}\left(\frac{T_{f,n}^{t}-T_{f,n}^{t-1}}{\Delta t}+u\frac{T_{f,n}^{t}-T_{f,n-1}^{t}}{\Delta z}\right)=ha_{s}\left(T_{s,n}^{t}-T_{f,n}^{t}\right),\left(1-\epsilon \right)\rho_{s}c_{p,s}\left(\frac{T_{s,n}^{t}-T_{s,n}^{t-1}}{\Delta t}\right)=ha_{s}\left(T_{f,n}^{t}-T_{s,n}^{t}\right),$$

applying the Euler method to two-phase energy conservation equations of PB sets a system of 2x2 equations. These equations depend on the temperature of the fluid and the bed for each node of space and time. This system can be easily solved because in each spatial and temporal node of fluid and rock, temperatures depend only on the conditions of the previous points, which are already known thanks to the initial and boundary conditions.

Furthermore, the explicit Euler method applied to the continuous solid phase model (two-phase- 1D) can be expressed as follows:

$$\epsilon \rho_{f}c_{p,f}\left(\frac{T_{f,n}^{t}-T_{f,n}^{t-1}}{\Delta t}+u\frac{T_{f,n}^{t}-T_{f,n-1}^{t}}{\Delta z}\right)=ha_{s}\left(T_{s,n}^{t}-T_{f,n}^{t}\right)+K_{f,eff,z}\frac{T_{f,n+1}^{t-1}-2T_{f,n}^{t-1}+T_{f,n-1}^{t-1}}{\Delta z^{2}}+Ua_{b}\left(T_{\infty }-T_{f,n}^{t}\right),\left(1-\epsilon \right)\rho_{s}c_{p,s}\frac{T_{s,n}^{t}-T_{s,n}^{t-1}}{\Delta t}=ha_{s}\left(T_{f,n}^{t}-T_{s,n}^{t}\right)+K_{\text{s,eff,z}}\frac{T_{s,n+1}^{t-1}-2T_{s,n}^{t-1}+T_{s,n-1}^{t-1}}{\Delta z^{2}},$$

and the explicit Euler method applied to the thermal gradient model in solid particles (two-phase- 1D):

$$\epsilon \rho_{f}c_{p,f}\left(\frac{T_{f,n}^{t}-T_{f,n}^{t-1}}{\Delta t}+u\frac{T_{f,n}^{t}-T_{f,n-1}^{t}}{\Delta z}\right)=K_{f,eff,z}\frac{T_{f,n+1}^{t-1}-2T_{f,n}^{t-1}+T_{f,n-1}^{t-1}}{\Delta z^{2}}+ha_{s}\left(T_{s,n}^{t}{|}_{\eta =d_{p}/2}-T_{f,n}^{t}\right)+Ua_{b}\left(T_{\infty }-T_{f,n}^{t}\right),$$

$$\rho_{s}c_{p,s}\frac{T_{s,n}^{t}-T_{s,n}^{t-1}}{\Delta t}=K_{s}\left[\frac{T_{s,n,\eta +1}^{t-1}-2T_{s,n,\eta }^{t-1}+T_{s,n,\eta -1}^{t-1}}{\Delta \eta^{2}}+\frac{2}{\eta }\frac{T_{s,n,\eta }^{t}-T_{f,n,\eta -1}^{t}}{\Delta \eta }\right].$$

#### 4.1.2 Implicit Euler method

The second variant of the Euler method is the implicit one. It consists of approximating the partial derivatives by backward increments of the function. The spatial and temporal increases of derivatives result in Eqs. (4)-(6) and the next additional equation:

$$\frac{\partial^{2}T}{\partial z^{2}}=\frac{T_{n+1}^{t}-2T_{n}^{t}+T_{n-1}^{t}}{\Delta z^{2}}.$$

The one-phase model can be rewritten in one dimension (one-phase -1D), using the implicit Euler method:

$$\left[\left(1-\epsilon \right)\rho_{s}c_{p,s}+\epsilon \rho_{f}c_{p,f}\right]\frac{T_{n}^{t}-T_{n}^{t-1}}{\Delta t}+\rho_{f}c_{p,f}\epsilon u\frac{T_{n}^{t}-T_{n-1}^{t}}{\Delta z}=K_{t,eff,z}\frac{T_{n+1}^{t}-2T_{n}^{t}+T_{n-1}^{t}}{\Delta z^{2}}+Ua_{b}\left(T_{\infty }-T_{n}^{t}\right).$$

The Schumann's model in one dimension (two-phase- 1D) applying implicit Euler method:

$$\epsilon \rho_{f}c_{p,f}\left(\frac{T_{f,n}^{t}-T_{f,n}^{t-1}}{\Delta t}+u\frac{T_{f,n}^{t}-T_{f,n-1}^{t}}{\Delta z}\right)=ha_{s}\left(T_{s,n}^{t}-T_{f,n}^{t}\right),\left(1-\epsilon \right)\rho_{s}c_{p,s}\left(\frac{T_{s,n}^{t}-T_{s,n}^{t-1}}{\Delta t}\right)=ha_{s}\left(T_{f,n}^{t}-T_{s,n}^{t}\right).$$

Like in the explicit Euler method, in these cases the FDM sets a system of 2x2 equations which can be solved because each spatial and temporal node depends on the conditions of the previous points only.

Finally, the implicit Euler method applied to the continuous solid phase model (two-phase- 1D) can be shown:

(8) $$\epsilon \rho_{f}c_{p,f}\left(\frac{T_{f,n}^{t}-T_{f,n}^{t-1}}{\Delta t}+u\frac{T_{f,n}^{t}-T_{f,n-1}^{t}}{\Delta z}\right)=ha_{s}\left(T_{s,n}^{t}-T_{f,n}^{t}\right)+K_{f,eff,z}\frac{T_{f,n+1}^{t}-2T_{f,n}^{t}+T_{f,n-1}^{t}}{\Delta z^{2}}+Ua_{b}\left(T_{\infty }-T_{f,n}^{t}\right),$$

(9) $$\left(1-\epsilon \right)\rho_{s}c_{p,s}\frac{T_{s,n}^{t}-T_{s,n}^{t-1}}{\Delta t}=ha_{s}\left(T_{f,n}^{t}-T_{s,n}^{t}\right)+K_{\text{s,eff,z}}\frac{T_{s,n+1}^{t}-2T_{s,n}^{t}+T_{s,n-1}^{t}}{\Delta z^{2}}.$$

This case cannot be solved directly, since the temperature at a point in the spatial and temporal node depends on the previous ones (known) and the neighbors (not known). As a novelty, Eqs. (10)-(11) shows the implicit Euler method applied to the thermal gradient model in solid particles (two-phase- 1D):

(10) $$\epsilon \rho_{f}c_{p,f}\left(\frac{T_{f,n}^{t}-T_{f,n}^{t-1}}{\Delta t}+u\frac{T_{f,n}^{t}-T_{f,n-1}^{t}}{\Delta z}\right)=K_{f,eff,z}\frac{T_{f,n+1}^{t}-2T_{f,n}^{t}+T_{f,n-1}^{t}}{\Delta z^{2}}+ha_{s}\left(T_{s,n}^{t}{|}_{\eta =d_{p}/2}-T_{f,n}^{t}\right)+Ua_{b}\left(T_{\infty }-T_{f,n}^{t}\right),$$

and

(11) $$\rho_{s}c_{p,s}\frac{T_{s,n}^{t}-T_{s,n}^{t-1}}{\Delta t}=K_{s}\left[\frac{T_{s,n,\eta +1}^{t}-2T_{s,n,\eta }^{t}+T_{s,n,\eta -1}^{t}}{\Delta \eta^{2}}+\frac{2}{\eta }\frac{T_{s,n,\eta }^{t}-T_{f,n,\eta -1}^{t}}{\Delta \eta }\right].$$

## 5 Solving finite differences

**Figure 9:**
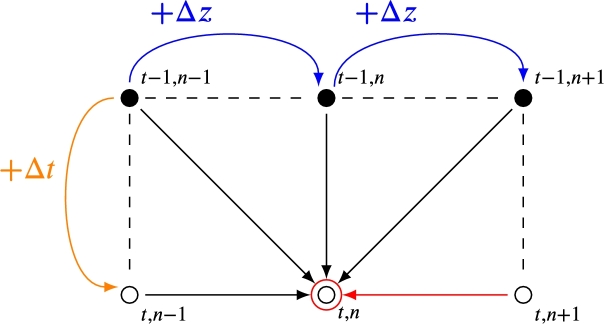
Algorithm to solve the explicit and implicit Euler method. Both methods require three previous temporal points and one previous spatial point to calculate the unknown temperature. In addition, the implicit Euler method requires one following spatial point. The main difference with the explicit method is highlighted with the red arrow in the scheme.

There are two resolution algorithms to solve the Euler method. An iterative algorithm is used for the explicit Euler method, and a matrix algorithm for the implicit Euler method. Fig. 9 graphically shows how, in an iterative algorithm, the temperature in one point depends on its previous neighbors. The earlier neighbors at the unknown point are well known. However, in the matrix algorithm, the temperature in one point depends on its previous neighbors, who are known, and the following ones, who are not known; this unknown point has a red arrow in Fig. 9.

### 5.1 Explicit Euler method. Iterative algorithm

The Explicit Euler method can be solved with the iterative algorithm because this finite difference method does not require the temperature of the future points, which are unknown. Note that the thermal conductivity factor is evaluated in a time before the actual point, this would be an error source solved by the matrix algorithm. The temperature of HTF and rock in each point can be directly solved using the solutions shown in B.

### 5.2 Implicit Euler method. Tridiagonal matrix algorithm

The implicit Euler method needs the temperature of future neighbors to the unknown point. Therefore, it is necessary to use the matrix algorithm. This algorithm can solve any model. For example, this algorithm is used to solve the continuous solid phase model equations in one dimension without any intermediate approximation. The first step starts from continuous solid phase model energy equations, Eqs. (8)-(9), where the implicit Euler method has been applied. These equations are expressed in tridiagonal arrays:

(12) $$T_{f,n}^{t}\alpha +T_{f,n-1}^{t}\beta +T_{f,n+1}^{t}\gamma +T_{s,n}^{t}\delta =T_{f,n}^{t-1}\epsilon +\eta ,$$

(13) $$T_{s,n}^{t}\alpha^{\prime }+T_{s,n-1}^{t}\beta^{\prime }+T_{s,n+1}^{t}\gamma^{\prime }+T_{f,n}^{t}\delta^{\prime }=T_{s,n}^{t-1}\epsilon^{\prime },$$

and coupling the temperatures of the fluid and the bed in the same array system:

$$MT^{t}=HT^{t-1}+B.$$

All parameters and constants used are detailed in C. Equation (14) represents the continuous solid phase model in one dimension, discretized by the implicit Euler method and compressed in one equation of tridiagonal arrays. The temperature of previous temporal points of the mesh are well-known, *i.e.*, $T^{t-1}$. However, the actual temporal mesh of spacial temperatures is unknown, i.e. the $T^{t}$ vector. A couple of $2N_{z}$ equations can be easily resolved using the reversed arrays. The matrix **M** groups the coefficients of $T_{f,s}^{t}$ in addition to the boundary conditions. The matrix **H** groups the coefficients of $T_{f,s}^{t-1}$. The matrix **B** includes the boundary condition for the fluid inlet temperature: $T_{f,inlet}$. The vector $T^{t}$ simultaneously groups the fluid and rock temperatures. All temperature spatial points of the actual time step can be easily obtained as follows:

(14) $$T^{t}=M^{-1}HT^{t-1}+M^{-1}B,$$

Initial conditions:

(15) $$T_{f,\forall n}^{1}=T_{s,\forall n}^{1}=T_{\infty }$$

Discretization of boundary conditions

(16) $$T_{f,1}^{\forall t}=T_{f,inlet},{\frac{\partial T_{f}}{\partial z}|}_{z=L}=0\overset{\frac{\partial T_{f}}{\partial z}=\frac{T_{n}^{t}-T_{n-1}^{t}}{\Delta z}}{\overset{︷}{⟶}}T_{f,Nz}^{t}=T_{f,Nz-1}^{t},{\frac{\partial T_{s}}{\partial z}|}_{z=0}=0\overset{\frac{\partial T_{s}}{\partial z}=\frac{T_{n}^{t}-T_{n-1}^{t}}{\Delta z}}{\overset{︷}{⟶}}T_{s,0}^{t}=T_{s,1}^{t},{\frac{\partial T_{s}}{\partial z}|}_{z=L}=0\overset{\frac{\partial T_{s}}{\partial z}=\frac{T_{n}^{t}-T_{n-1}^{t}}{\Delta z}}{\overset{︷}{⟶}}T_{s,Nz}^{t}=T_{s,Nz-1}^{t}.$$

## 6 Selecting the physical model and the solving method

### 6.1 Models overview

In this section, a two-step procedure is followed. First, the results from several physical models and a fixed-solving method are compared. Afterward, a brief sensitivity analysis of some key parameters is shown. Finally, a condensed table comparing computation time for the considered solving methods is shown.

First, the implicit Euler method is applied to several models commented on in Sec. 3. It is considered a cylindrical deposit with a 1.8 m length, and internal diameter of 0.584 m, containing alumina beads with 8 mm diameter particles as in the experimental work by Cascetta et al. [80]. The heat transfer fluid is air. A detailed table with all the required parameters is presented in App. E. These experimental conditions are considered for a first overview purpose because, in principle, even simple physical models should properly reproduce experiments in those conditions.

**Figure 10:**
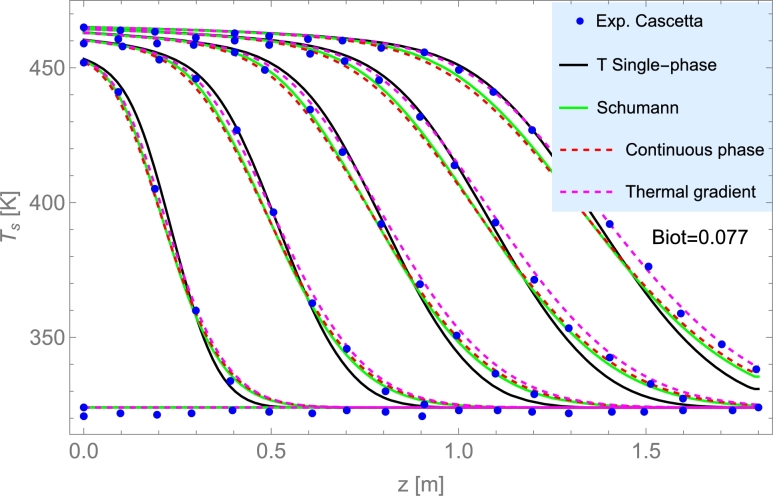
Comparison against the experimental results by Cascetta et al. [80] of the numerical results from some theoretical models. Total charge time, *t*_*charge*_, is 4129 s. Curves cover the interval from *t* = 0 to *t*_*charge*_ with a time step *t*_*charge*_/5. More details in Subsec. 8.1. The thermal gradient model is computed with 30 internal layers in each particle.

Fig. 10 displays the evolution of the solid temperature in real units against the height of the layers for the following physical models mentioned above: single phase, Schumann, continuous solid phase, and thermal gradient inside particles model. The experimental curves at different times, obtained from [80], are also displayed. As is observed in the figure, all simulated curves correctly reproduce the experimental evolution. The good agreement with experiments followed in the figure for single phase and Schumann's model only is valid when axial effective thermal conductivities are small or when the heat capacity of the bed is larger than that of the fluid, as commented in Secs. 3.1 and 3.2.1. Continuous solid phase and thermal gradient models can reproduce the experimental behavior on a broader interval of conditions as will be commented below. However, it requires a greater computational effort (next section). For experimental situations similar to those in Fig. 10, even the simplest models include the main physical ingredients to reproduce tests.

For instance, single phase model does not match experimental results when HTF velocity increases since the convection by axial flow term acquires more weight in the equations, dissociating the temperatures between both phases. Therefore, this model can be discarded to ensure a wide range of applicability on fluid velocities. Schumann's model is a good model due to low $K_{\text{s,eff,z}}$ (PB is composed of spherical particles). However, when energy is stored for large times, it is necessary to include thermal conductivities in HTF and storage material due to thermocline degradation because of heat conduction, this will be explained in Sec. 11. An excellent indicator for selecting the physical model in a particular problem is the Biot number, defined as:

$$Bi=\frac{h\cdot d_{p}}{K_{s}},$$

where $d_{p}$ is the particles diameter, *h* the convection coefficient and $K_{s}$ the solid particles' thermal conductivity. Small *Bi* numbers generally arise from small particles in the bed or high solid conductivities (about Bi<0.5). In these cases, the single phase and Schumann's model are appropriate. Otherwise, a continuous solid phase model can be applied, and for large particles in the bed, the thermal gradient model should be more accurate. Next, a condensed sensitivity analysis considering continuous solid phase and thermal gradient is shown.

**Figure 11:**
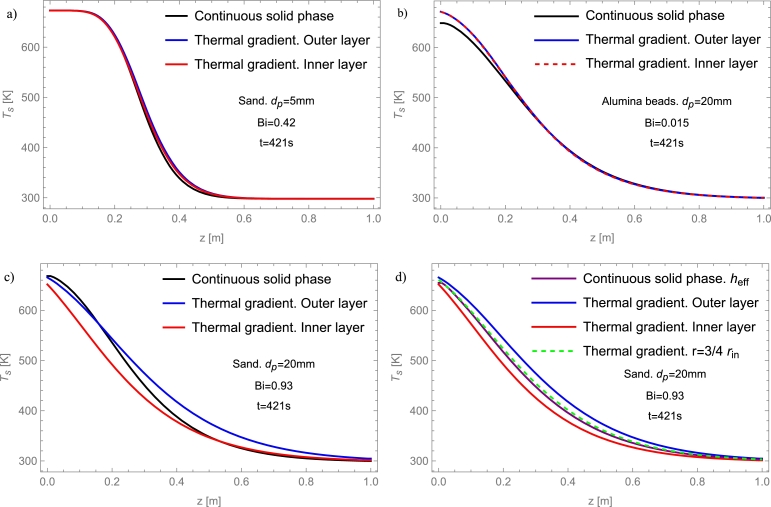
Influence of Biot number and *h*_*eff*_ in continuous solid phase model and thermal gradient inside particles model. a) When the Biot number is small, due to low particle *d*_*p*_, the continuous solid phase model and the thermal gradient inside the particles model converge to the same results. Also, the inner and outer layers of the gradient model converge. b) When the Biot number is small, due to high storage material thermal conductivity (*K*_*s*_), the continuous solid phase model and the thermal gradient inside particles model also converge to the same results. Note that alumina as storage material is only used in this figure. c) For a high Biot number, it can be seen the divergence in temperature solutions from both models. Also, it can be noticed the gradient temperature inside the storage material particles. d) For a high Biot number, when an effective heat convection coefficient (*h*_*eff*_) is used, it can be seen the convergence between models for an average internal layer.

In D are summarized all parameters used in numerical simulations of Fig. 11. A typical packed-bed system of sand and air has been chosen, with 1 meter long. Maximum temperature is 400 °C and minimum is 25 °C. The mass flux ratio is 0.5 kg/s. Figs. 11(a) and 11(b) show small Biot number situations, *i.e.*, Bi<0.5, due to low $d_{p}$ (sand) or high $K_{s}$ (alumina). Curves from both models converge, showing the equivalence between them. Nevertheless, for large Biot numbers, methods do not yield close results. This is shown in the lower panels of Fig. 11 for a Biot number of 0.93. It is just an academic case for completeness because, in practical PB systems, HTF needs to raise its temperature in discharge mode thanks to quickly cooling of storage particles from inside to outside.

Sometimes, an effective convection coefficient [98] is used for large Biot numbers, avoiding the direct use of the thermal gradient model itself, which computationally is more expensive than the two-phase continuous model. This effective convection coefficient is defined as,

(17) $$h_{eff}={\left(\frac{1}{h}+\frac{d_{p}}{10K_{s}}\right)}^{-1}.$$

In Figs. 11(c) and 11(d), a comparison between the results from the strict thermal gradient model (red curve in Fig. 11(c)) and the continuous solid phase model considering $h_{eff}$ is conducted (red curve in Fig. 11(d)). Differences are insignificant, and so the latter constitutes a reasonable approach when the thermal gradient inside particles is not negligible and computational time is to be saved.

### 6.2 Solving methods comparison and computing times

**Figure 12:**
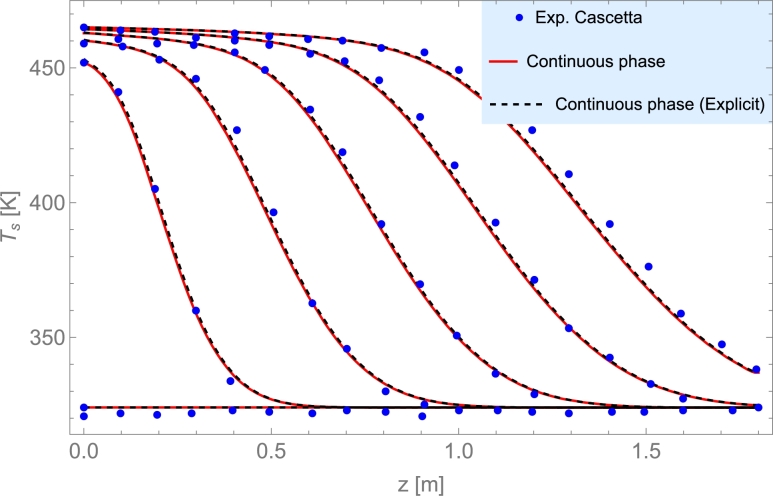
Continuous solid phase model solved through explicit and implicit Euler. Both methods have equal results for low effective thermal conductivities in storage material.

Explicit and implicit Euler methods for solving the energy sets of equations yield similar results provided that conductivities (for solid and fluid phases) are small or when they are a priori neglected (as in the case of considering one-phase or Schumann's models). This fact is shown in Fig. 12 where the temperatures of the solid and the fluid obtained with explicit and implicit Euler's schemes are displayed. For storage materials composed of small individual particles, bed effective conductivities usually are small, so differences from the predictions of both computational schemes are similar. Nevertheless, as conductivity increases, the explicit method loses accuracy as it uses a later evaluation of thermal conductivity.

The computation time is a key ingredient when selecting a particular physical model and a solving scheme. Ismail et al. [97] performed a comparative analysis of the computation times for several methods. An in-house summary table with computing times normalized to the smallest one (one phase model and implicit Euler method) is presented in Table 7 for a particular case. When specific parameters are changed, numerical values could slightly vary, but relative differences remain almost unchanged. Apart from the comments related to the Biot number and thermal conductivities, Table 7 shows that the two-phase continuous solid model, solved through an implicit Euler algorithm, has lower computational cost than the equivalent explicit method, which is a definitive major asset.

**Table 7:** Normalized computing times with the one-phase model, which exhibits the smallest computing time.

Model	$\frac{\mathrm{Computing time}}{\mathrm{One phase model time}}$
Single or one phase model	1
Schumann's model	2.4
Continuous solid phase model (implicit Euler)	2.8
Continuous solid phase model (explicit Euler)	4.8
Thermal gradient (15 layers)	9.6
Thermal gradient (30 layers)	31
Thermal gradient (40 layers)	57.2

The direct consideration of the thermal gradient in the particles rapidly increases computation times. In addition, many internal layers must be used to obtain good precision in solutions. Another interesting conclusion is that, with a great difference, the single-phase model is faster, but its application is limited to the cases commented on before. The continuous solid phase solved through the Euler implicit method is not much more expensive than Schumann's model but has a wider applicability.

From our viewpoint, the continuous solid phase model solved with an implicit Euler algorithm constitutes a good compromise between computing time and versatility and reliability for solving the systems dynamics as those commented in the Introduction: A-CAES (and possible derivations), PTES and CSP. It is difficult to give a definite numerical estimation of the time required to solve the dynamics of any of those systems for a certain number of cycles. It will depend on the peculiarities of system configuration and the considered constraints. And also, of course, of the objectives of the work and the importance of cycle-to-cycle dynamics to achieve those aims.

**Figure 13:**
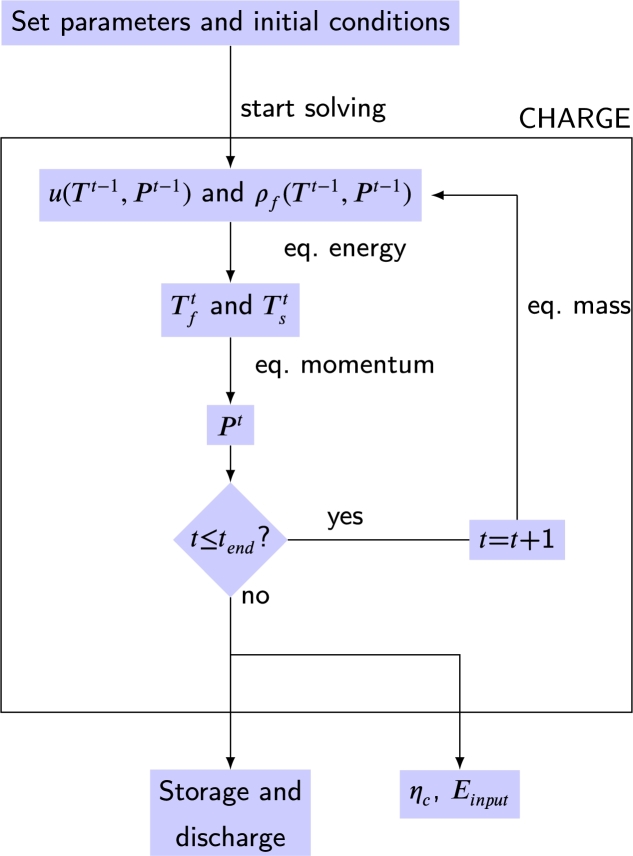
Mathematical model algorithm. Solved by the implicit Euler method and taking into account pressure drops, see Sec. 9.

Thus, all the subsequent results shown in this paper are obtained (otherwise, it is explicitly mentioned) with the continuous solid phase model (1D) using the implicit Euler algorithm. The effective fluid-rock convective coefficient as defined in Eq. (17) is included. Moreover, an effective bed density, $\rho_{\text{eff}}$, is also taken into account in the model, intending to account for wall effects on energy storage in PB performance (see Eq. (2)). Fig. 13 shows a diagram of the general resolution algorithm for the proposed model, including pressure drops, as it will be detailed in Section 9. From now on, only this model will be used to solve the PB systems of each section.

## 7 Influence of spatial and temporal step

To ensure the independence of the solution from the chosen discretization mesh, it is necessary to study the influence of spatial and temporal steps in the selected resolution method. There is a trade-off between computing time and mesh refinement, the former will increase exponentially with the latter.

### 7.1 Influence of spatial step

**Figure 14:**
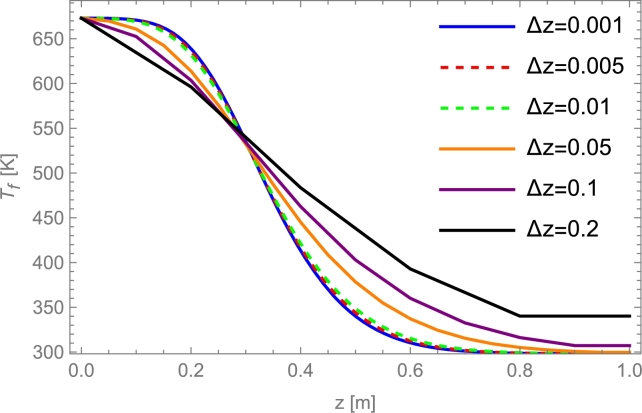
Thermal profile of PB as a function of spatial step size. The lower spatial step will give us greater accuracy in the solutions. All curves are plotted at t=500 s. Value of Δ*t* = 1 s.

Parameters used in the simulation are summarized in App. D. To show the influence of the spatial step on the bed temperature profile, a few refinements of the spatial mesh are compared; this is depicted in Fig. 14, where a loss in precision can be seen as the spatial intervals increase.

**Figure 15:**
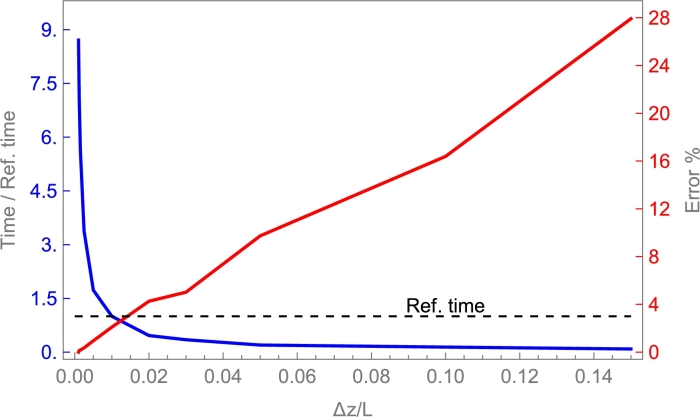
Normalized computing time (blue) and error solution (red) as a function of spatial step size. Reference spatial step is set at Δ*z*/*L* = 0.01.

The trade-off mentioned above can be seen in Fig. 15. This figure compares the computing time for the case Δz/L=0.01 (blue curve in Fig. 15), which is used as a reference to normalize the time (arbitrarily used as a reference for illustrative purposes). Notice that increasing the spatial step size beyond this value will not significantly improve computing time. On the other hand, the relative deviation with respect to the case of maximum resolution, $T^{⁎}$, obtained with Δz/L=0.001, at t=500 s and z=0.5 m, that is,

$$\delta T=\frac{T-T^{⁎}}{T^{⁎}}\times 100\text{\%},$$

this curve shows that Δz/L=0.01 has a significant deviation. Notice that this is a definition of the error *δT* at a point where it will be maximum; that is, it will be the maximum error. Other errors can also be defined, such as the average. A good compromise can be found at a value of Δz/L=0.0025 since smaller values do not represent a significant improvement in the calculations compared to the very high computational cost that does involve further reducing the time step.

### 7.2 Influence of temporal step

**Figure 16:**
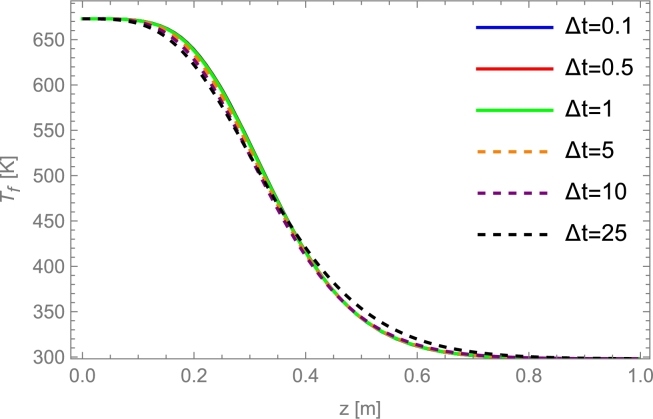
Thermal profile of PB as a function of time step size. All curves are plotted at t=500 s. Value of Δ*z*/*L* = 0.01.

**Figure 17:**
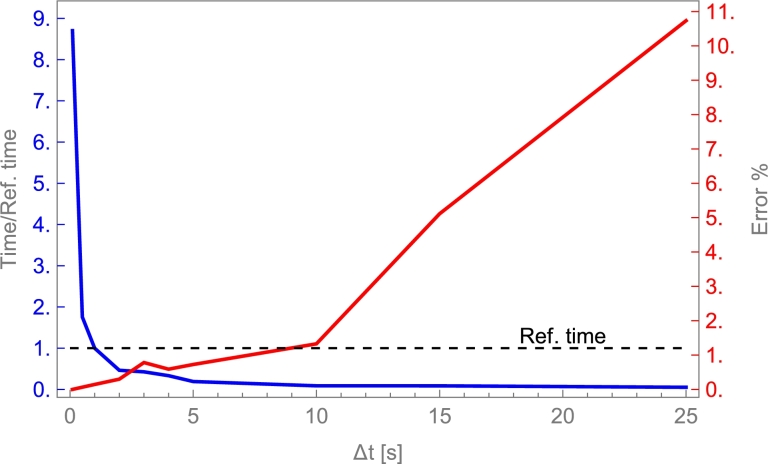
Normalized computing time (blue) and error solution (red) as a function of time step size. The reference time step is set at Δ*t* = 1 s.

Fig. 16 shows a slight temperature variation, changing the time step. All curves are plotted at t=500 s. For the analysis of Δ*t*, in Fig. 17 Δt=1s is a reference for the normalization of the computing time. For the error, the smallest value of Δ*t*, considered the most precise, is used as a reference. A step value of Δz/L=0.01 is used to reduce computing time without raising significantly the error. Fig. 17 shows that a time step of one second is a good decision; reducing it does not imply a considerable improvement in the error but does lead to a very high computational cost. Increasing the spatial step to 5 seconds means an error of 1% but a reduction in computing time by a factor of 5.

## 8 Model validation

The continuous solid phase model in one dimension solved by the implicit Euler method has been selected for solving the PB system due to its precision and low calculation time. Next, the results predicted from this simulation scheme are compared with various experiments.

### 8.1 Cascetta [80]

**Figure 18:**
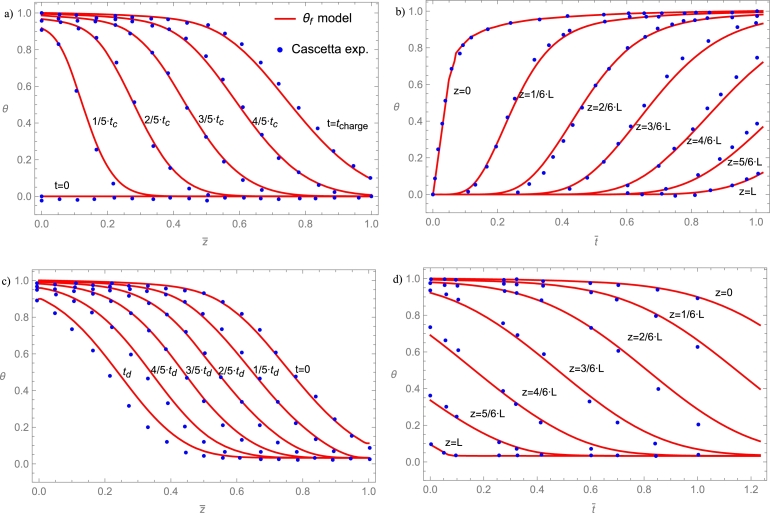
Comparison between the model and Cascetta et al. experimental set rig [80]. a) Charge: temperature profiles of PB as a function of position. Various moments from the initial time to the final time of the charge are shown, with increases of *n*/5 ⋅ *t*_*charge*_. b) Charge: temperature profiles of PB as a function of time. Various positions are shown from the initial position to the final position, increases of *n*/6 ⋅ *L*. c) Discharge: temperature profiles of PB as a function of position. Various moments from the initial time to the final time of the discharge are shown, increases of *n*/5 ⋅ *t*_*discharge*_. d) Discharge: temperature profiles of PB as a function of time. Various positions are shown from the initial position to the final position, increases of *n*/6 ⋅ *L*.

The proposed numerical model is compared with the experimental results obtained in 2016 [80]. It is an air-alumina beds experiment whose boundary condition for the inlet air is not constant. Its temperature increases up to a maximum value. So, it is used as a variable condition for $T_{f,inlet}$. All parameters of the experiment are specified in Appendix E. Results of the model successfully fit the experiments as shown in Figs. 18(a)-18(d). Table 8 shows some more quantitative values, which include the validation of [72]. The dimensionless quantities of Eqs. (18)-(20) are considered.

(18) $$\theta =(T-T_{min})/(T_{max}-T_{min}),$$

(19) $$\overset{¯}{z}=z/L,$$

(20) $$\overset{¯}{t}=t/t_{max}.$$

In Eq. (20), $t_{max}$ refers to maximum time to charge the PB until θ(z=L)=0.1 or discharge PB until θ(z=0)=0.9.

**Table 8:** Quantitative analysis of validation. Charge and discharge comparison between the numerical model considered in this work, [80] and [72].

Author	*t*_*charge*_	Absolute	Relative	Author	*t*_*discharge*_	Absolute	Relative
	(s)	Error (s)	Error (%)		(s)	Error (s)	Error (%)
Cascetta [80]	3985	−−	−−	Cascetta [80]	2700	−−	−−
Esence [72]	4245	260	6.6	Esence [72]	2795	95	3.5
This paper	4129	144	3.6	This paper	2513	187	6.9

As shown in the Figures and the table, the comparison between experiments and simulations is reasonably good. As it was commented in Sec. 6.1 simpler physical models such as single phase and Schumann's could reproduce experiments in this case.

### 8.2 Izquierdo [108]

**Figure 19:**
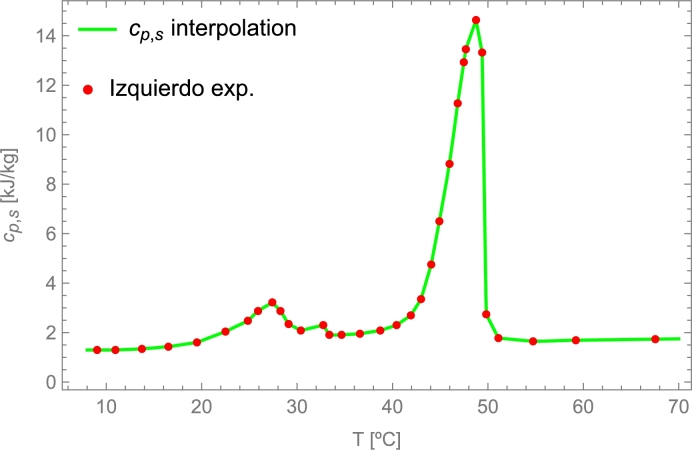
Interpolation from experimental measures of GR-50 *c*_*p*,*s*_. The temperature-dependent interpolation of *c*_*p*,*s*_ is used directly in the mathematical model of the continuous solid phase model. Experimental data from [108].

**Figure 20:**
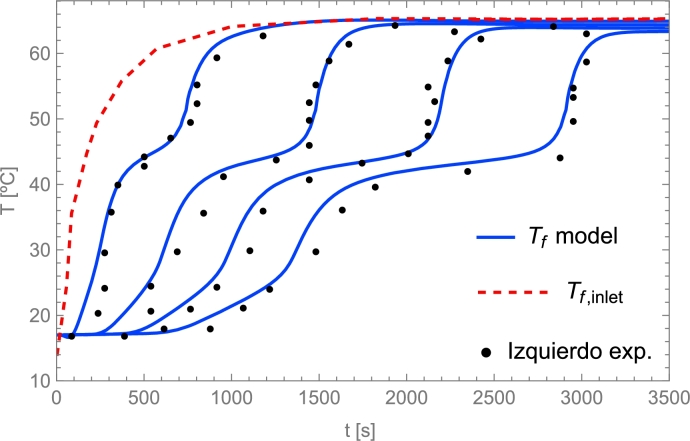
Validation of the model using experimental data from [108]. Temperature curves from left to right are plotted at *z* = 0 (temperature inlet at plenum in red color), *z* = 0.025, 0.075, 0.125, 0.175 m (blue color).

This experimental setup, which uses a bed with a PCM material, is considered to check the model's validity for latent heat processes. The experimental setup comprises air-PCM with a storage height of L=0.2 m. The storage material is Rubitherm GR-50 ©. The heat capacity of GR-50 was measured by Izquierdo [108], see Fig. 19. An interpolation will be used directly to calculate the bed's heat capacity as a temperature function at each spatial and temporal step. The details of the experiment are described in Appendix E. Applying a variable $T_{f,inlet}$ for the fluid inlet into the bed (z=0), the numerical model gives reasonably good results (see Fig. 20).

### 8.3 Xie [73]

The experiment of [73] is replicated. This validation pretends to extend the validation of the numerical model for liquid HTF, in this case, water. In this way, the numerical model will be validated for liquid as well as gaseous fluids. The experiment consists of a soda-lime glass tank as storage material and water as HTF. The main problem of this experiment is the absence of a diffuser in Case 1 of [73], which is the only one that presents the bed temperature profiles. The lack of the diffuser creates HTF jets in the axial axis. Therefore, the experimental measurements of the thermocouples in the axial axis present a temperature higher than the temperature of the numerical model.

**Figure 21:**
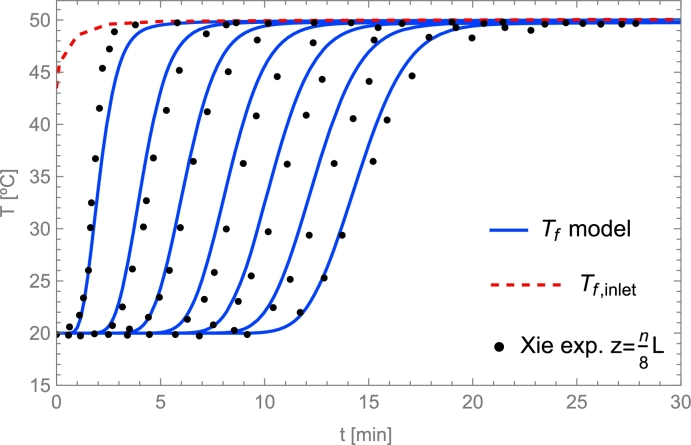
Validation using experimental data from [73]. Temperature curves from left to right are plotted from *z* = 1/8*L* = 0.05 m to *z* = 7/8*L* = 0.35 m.

Furthermore, the temperature profile $T_{f,inlet}$ is not specified, so it must be deduced from the temperature profile of the following thermocouple. This deduction consists of applying a time lag *τ*, as explained by [72] and shown in Eq. (21). It is an assumption that implies a low degradation of the thermal front in the bed space covered. The results are quite satisfactory, see Fig. 21. The specific data of the experiment are shown in App. E.

(21) $$\tau ={\left(\frac{\frac{\epsilon \rho_{f}c_{p,f}}{\epsilon \rho_{f}c_{p,f}+(1-\epsilon )\rho_{s}c_{p,s}+x_{w}\rho_{w}c_{p,w}}\cdot u}{l}\right)}^{-1},$$

where *l* is the length between $T_{f,inlet}$ and the measure of the first thermocouple.

### 8.4 Meier [75]

**Figure 22:**
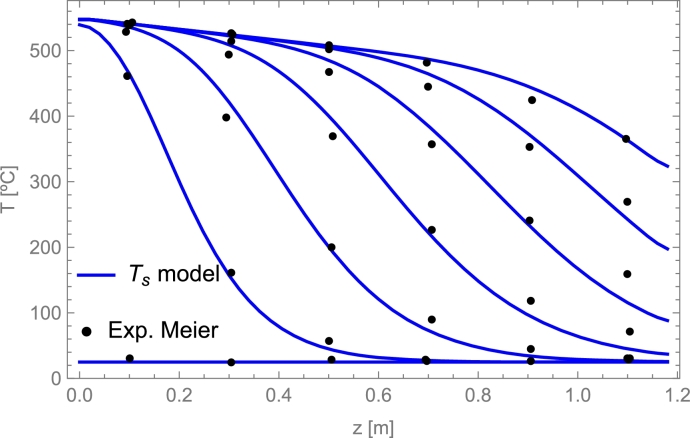
Temperature profiles of the numerical model compared with the experimental setup of Meier et al. Specifically data from the ARIANE experiment [75]. Temperature profiles are plotted from left (*t* = 0) to right (*t* = 3 h) increasing 0.5 h.

To validate the model at high temperatures, it is necessary to reproduce an experiment like Meier et al., called ARIANE [75]. It is an experimental setup with a working fluid inlet temperature of 550 °C. It is one of the few setups that operates at such high temperatures in the literature. The bed material comprises magnesium silicate spheres confined in a steel cylinder. The charge compressor operates with a pressure close to ambient (1 atm), and the bed suffers practically negligible pressure drops, around 100 Pascals. The details of the experiment are shown in Appendix E. The good validation of the numerical model compared with the experimental setup is shown in Fig. 22. Therefore the proposed numerical model effectively reproduces PBs with high and low temperatures, with latent and sensible heat storage materials, and with liquid or gaseous working fluids (HTF).

## 9 Pressure drops

Momentum equations for porous media considering viscosity and drag forces that provoke resistance to the movement of the fluid through the solid have been proposed in the literature [109], [110]. Nevertheless, the most usual way to estimate pressured drops in the case of gaseous fluids is the empirical formula developed by Ergun [111] a long time ago. It depends on viscosity, velocity, and void fraction in the following way:

$$\frac{-\Delta P}{L}=150\frac{{\left(1-\epsilon \right)}^{2}}{\epsilon^{3}}\frac{\mu_{f}}{d_{p}^{2}}u+1.75\frac{\left(1-\epsilon \right)}{\epsilon^{3}}\frac{\rho_{f}}{d_{p}}u^{2}.$$

To close the numerical algorithm, it is necessary to use the mass conservation equation:

$$\frac{\partial \epsilon \rho_{f}}{t}+\overset{\to }{\nabla }\cdot (\rho_{f}\overset{\to }{u})=0.$$

Fig. 13 shows the algorithm of the numerical model to solve the PB equations and how pressure drops are taken into account in each time cycle and in each spacial point.

**Figure 23:**
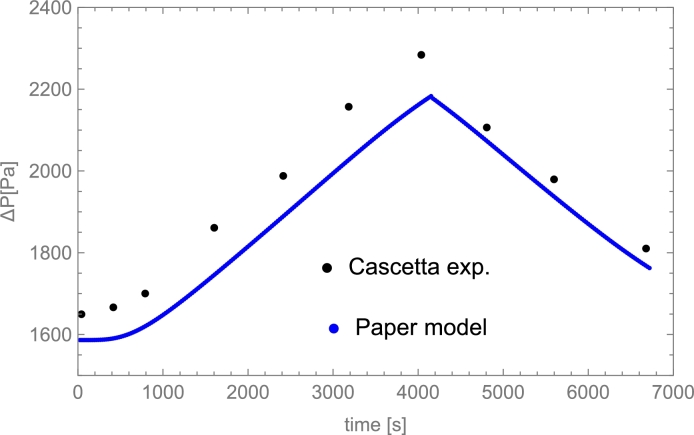
Pressure drops as a function of time for charge and discharge. Results from the computational model are compared against the experiments from Cascetta et al. [80].

Fig. 23 shows the validation of the numerical model in pressure drop losses. The experimental pressure drops of Cascetta et al. [80] with the model are compared, finding a good fit.

## 10 Stored energy and performance

This section shows how to calculate the energy stored and recovered from the bed and how to calculate the storage efficiency. According to [112], the stored energy and the maximum energy that can be stored are defined as follows:

$$E_{stored}=\int_{0}^{L}A_{t}[\int_{T_{s}\left(t=t_{0}\right)}^{T_{s}\left(t=t_{charge}\right)}\left(1-\epsilon \right)\rho_{s}c_{p,s}dT+\int_{T_{f}\left(t=t_{0}\right)}^{T_{f}\left(t=t_{charge}\right)}\epsilon \rho_{f}c_{p,f}dT]dz,E_{stored}^{max}=\int_{0}^{L}A_{t}[\int_{T_{s}\left(t=t_{0}\right)}^{T_{max}}\left(1-\epsilon \right)\rho_{s}c_{p,s}dT+\int_{T_{f}\left(t=t_{0}\right)}^{T_{max}}\epsilon \rho_{f}c_{p,f}dT]dz.$$

In these integrals, variables are temperature and position, and densities and heat capacities are temperature-dependent. Furthermore, temperature varies with the position of the bed as there is a temperature gradient. The integrals are solved numerically with the trapezoid rule.

The energy introduced during charging through the fluid is $E_{input}$. There is also a loss of outgoing energy during charging, as the hot fluid escapes through the bottom of the bed: $E_{output}$. In addition, the energy recovered during the discharge is defined as $E_{recovered}$.

$$E_{input}=\int_{0}^{t_{charge}}\int_{T_{f}(t=0)}^{T_{in}}\epsilon A_{t}\rho_{f}c_{p,f}u\;dTdt,E_{output}=\int_{0}^{t_{charge}}\int_{T_{f}(t=0)}^{T_{out}}\epsilon A_{t}\rho_{f}c_{p,f}u\;dTdt,E_{recovered}=\int_{0}^{t_{discharge}}\int_{T_{in}}^{T_{out}}\epsilon A_{t}\rho_{f}c_{p,f}u\;dTdt.$$

Finally, the charge, discharge, and total efficiency of packed-bed storage are defined as:

$$\eta_{charge}=\frac{E_{stored}}{E_{input}-E_{output}},\eta_{discharge}=\frac{E_{recovered}}{E_{stored}},\eta_{total}=\frac{E_{recovered}}{E_{input}-E_{output}}=\eta_{charge}\eta_{discharge}.$$

**Figure 24:**
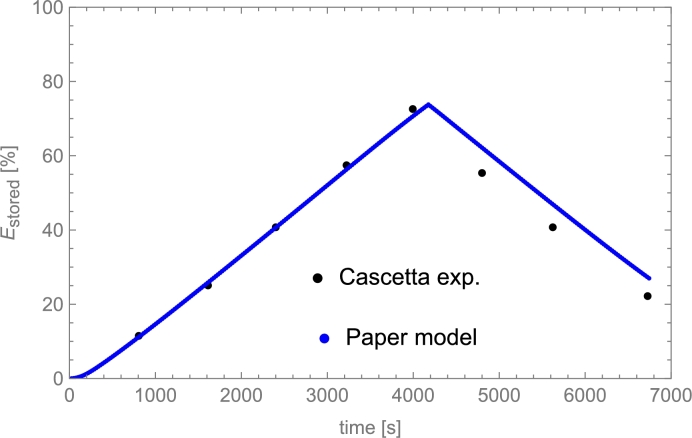
Stored energy as a function of time for charge and discharge. Stored energy in percentage (%) is defined as $E_{stored}/E_{stored}^{max}$.

Fig. 24 shows the validation of the numerical model in energy storage calculations. The numerical model is compared with experimental results in [80]. The results are satisfactory.

When considering PB thermal storage in a certain plant (A-CAES, PTES, etc.), working on cycles, a study should be done to determine the optimal charge in the PBs, which perhaps should not be too high to avoid undesirable losses. Thus, in overall plant optimization analyses, PBs charge could be considered an optimization parameter.

## 11 Analysis of performance and energy losses over time

A study for a PB system in similar conditions to the experimental setup of Cascetta et al. [80] is presented in this section. This system is widely validated since it has obtained the temperature profiles for successful charges and discharges (see Figs. 18(a)-18(d)), the pressure drops (Fig. 23) and the energy stored in the system (Fig. 24).

First, to know the influence of the variation of air mass flow rate on the performance of the packed-bed, the bed is submitted to several progressive discharges with different mass flow rates. In all cases, the charge is equal. The response of the bed to this kind of situation is interesting for applications that require repeated charge-discharge cycles, for instance, in PTES technology relying on Brayton cycles (see Sec. 1.2). Table 9 shows the results of recovered energy and discharge cycle efficiency for the experimental results of Cascetta et al. [80], the numerical model considered in this work for the same discharge fluid velocity as Cascetta and finally the numerical model for different fluid velocities. Energy storage in the wall is considered. The charge is assumed constant and equal to that carried out by Cascetta. The fluid velocity in discharge varies from half to 32 times more. For the base case, the upper part of Table 9 shows a good fit for the experimental results. By reducing the speed by half, the same efficiency is obtained. As the fluid velocity increases, efficiency decreases since the fluid needs more time to thermalize with the storage material and the mass flow rate is high. In addition, if fluid velocity increases, the energy extracted from the bed wall is reduced. The reduction in efficiency is not negligible. From the results in Table 9, it should also be concluded that although, in principle, ranges for fluid velocities are large, pressure decays limit them. They are too large at high speeds.

**Table 9:** Influence of working fluid discharge velocity on bed efficiency. Energy storage in wall is considered. In all cases *E*_*input*_ = 30.08 kW⋅h and *E*_*storage*_ = 30.07 kW⋅h, and *η*_*charge*_ = 0.99, except for the case of Cascetta, where *η*_*charge*_ = 0.95.

	*E*_*recovered*_	*η*_*discharge*_	*η*_*total*_	charge	discharge	Δ*P*
	[kW⋅h]			time [s]	time [s]	[Pa]
Cascetta	−−	0.70	0.66	3985	2700	1600-2290
Numerical model	18.74	0.62	0.62	4129	2513	1586-2183

0.5*u*_*discharge*_	18.70	0.62	0.62	4129	5010	455-642
2*u*_*discharge*_	18.45	0.61	0.61	4129	1240	5879-7958
4*u*_*discharge*_	17.87	0.59	0.59	4129	604	22585-30288
8*u*_*discharge*_	17.00	0.57	0.57	4129	291	88475-118060
16*u*_*discharge*_	15.80	0.53	0.53	4129	138	350173-466057
32*u*_*discharge*_	14.64	0.49	0.49	4129	65	(1.39-1.85)⋅10^6^

On the other hand, this same study notes that decreasing the discharge fluid velocity reduces pressure drops. Although there are acceptable margins, pressure drops must be considered to model a complete Brayton-type thermodynamic circuit.

**Figure 25:**
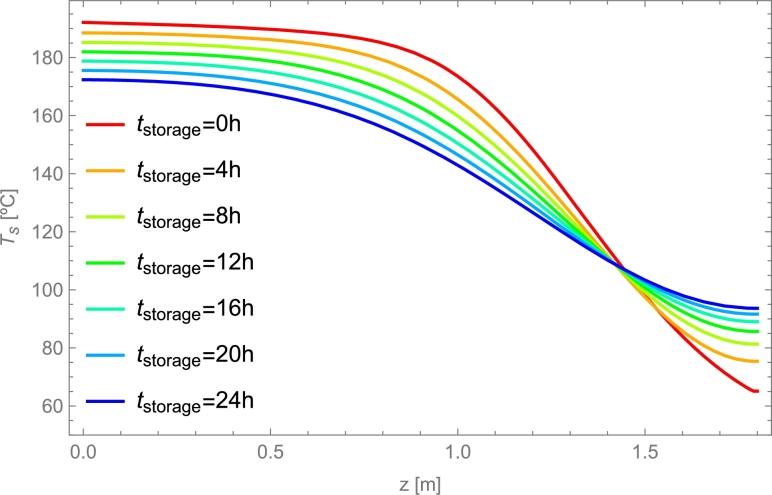
Bed temperature profiles over time spent in energy storage.

The second study carried out refers to the energy loss in the PB during the static storage time. As Table 10 shows, the energy of the bed will be reduced due to heat leaks during the storage hours. Likewise, in Fig. 25, an evident degradation of the thermocline of the bed is observed as the hours of storage pass. It is worth highlighting the few thermal energy losses suffered by the system despite the hours of storage considered. The same discharge condition as Cascetta et al. is used; that is, the fluid outlet temperature must be greater than 90% of the dimensionless temperature of the system. For the charge carried out, it is possible to recover the stored energy according to the discharge criterion until approximately 17 hours.

**Table 10:** Influence of storage time on bed efficiency.

storage	discharge	*η*_*discharge*_	*E*_*input*_	*E*_*recovered*_	*E*_*loss*_
time [h]	time [s]		[kW⋅h]	[kW⋅h]	[kW⋅h]
0	2513	0.62	30.07	18.74	0.00
5	2145	0.52	30.07	15.58	0.95
10	1669	0.39	30.07	11.81	1.88
15	889	0.20	30.07	6.12	2.79
16	609	0.14	30.07	4.16	2.96
17	79	0.017	30.07	0.51	3.14

## 12 Summary and conclusions

The present work is an updated guide for analyzing packed-bed storage systems for different applications, some of which have been strongly boosted in the last few years. Its main aim is to serve as a practical guide for researchers developing different large-scale thermal energy storage technologies.

In the Introduction, a summary of the work performed by several research groups in the last years is condensed. Novel applications being developed are described, such as high-temperature concentrated solar power, pumped thermal energy storage, compressed or liquid air energy storage, and others. The work done during the last years and the packed-bed systems' role in large-scale thermal energy storage systems are discussed.

On the other hand, different physical models based on the resolution of energy and mass conservation equations with analytical or numerical approaches are exposed, from the simplest models (one-phase, Schumann's) adequate for basic calculations on particular conditions to more refined ones valid for gas or liquid HTFs, sensible or latent storage, and intermediate or high temperatures. All of them are explained comprehensively, highlighting the most determinant physical parameters in each case. For non-experts, finite differences methods for the computational calculation of thermoclines are described in detail. Particularly, explicit and implicit Euler methods in one dimension are explicitly formulated. Comparison among the predicted numerical results for several physical models and different numerical solution schemes is done. Also, a discussion on considering the Biot number as a key parameter for deciding which model can be adequate to solve a particular case is performed.

The continuous solid phase model solved through the implicit Euler algorithm is a good choice in various situations. The influence of the time and spatial steps on the computing time and the expected precision of the solutions is accomplished. The spatial step is analyzed per unit of bed length, and the temporal step is analyzed in actual units. The spatial step should be Δz≤0.0025L, to achieve an acceptable precision in the error and a relatively low computing time. The time step can be set to 1 second to achieve very low error and moderate speed. However, time steps of the order of 5 seconds significantly speed up computing time at a relatively low error cost. So, it can be used to compute large-duration storage, as it occurs in new thermal storage applications.

The 1D two-phase continuous solid model is successfully validated against the experimental work from several authors for high and low temperatures, sensible and latent heat storage materials, and liquid or gaseous working fluids such as HTF. The resolution of the energy conservation equations is validated to obtain the temperature profile, bed pressure drops, stored energy, and system performance.

A particular deep study of a base case previously validated, a PB working with alumina beads and using air as HTF, is shown. The study analyzes the influence of the fluid velocity on the discharge and the influence of the steady storage time of the bed. In the study of fluid velocity, it is deduced that an increase in discharge fluid velocity implies a decrease in performance and a significant increase in pressure drops. However, large ranges of download velocities are acceptable. They also reduce the time required to discharge the bed.

In the study of the storage time, it is deduced that the energy lost over the hours is not very high. Still, there is a considerable degradation of the thermocline, which affects the overall system's efficiency. However, storage times of hours are within acceptable limits for this test rig, which has only 100 mm of mineral wool insulating layer. The insulating capacity of the system to reduce losses has room for improvement.

Systems with different configurations can be studied in the future to reduce the effect of thermocline degradation throughout the hours of storage. For instance, this line of action is proposed by [64], where the bed alternates layers of material storage with layers of air to reduce the axial thermal conductivity between layers and maintain the high thermocline for more hours. Another interesting configuration is proposed in [113], where a PB with radial flow is developed instead of axial flow. This configuration shows a reduction in pressure drops through the PB section.

In summary, we have presented an updated review of thermal energy storage based on packed-bed systems for their applications in current technologies for producing renewable energy. Avoiding an excessively technical presentation, the physical mechanisms underlying heat transfer dynamics in these systems were shown. Also, the most usual numerical methods for resolving the sets of partial differential equations appearing in these problems were comprehensively explained. Various experimental results for sensible and latent heat modes were shown with the corresponding simulated thermoclines to give the reader a global vision of the state-of-the-art.

As condensed in this review, packed-bed systems applied to energy storage technologies are full of life. Very active research is being done in mechanical and thermal large-scale energy storage systems such as A-CAES, AA-CAES, LAES, and other variants, high-temperature concentrated solar power, and pumped thermal storage systems. PBs play a key role, and several research lines are open for the immediate future. These research directions are at least twofold. First, to deepen the packed-bed systems' performance and second, their integration into the mentioned technologies.

Within the first point, research is focused on finding materials (for sensible and latent heat) susceptible to improve efficiency but affordable from economic and environmental viewpoints, capable of being operated during many cycles without degradation and, of course, with good thermal properties. As commented above, another open point is minimizing pressure decay in PB dynamics. Moreover, optimization analysis involving in a simultaneous form container size and shape parameters, void fraction, bed particle size, etc., together with bed materials and HTF properties, is also appealing.

Concerning the integration of PBs in large-scale thermo-mechanical energy storage, there are challenges in all the technologies mentioned above. Globally, interesting research directions should incorporate the integration of PBs in systems operating for a long number of cycles with possibly different charging/storage/discharging periods. The performance of whole plants under different time requirements is highly stimulating to widen the possible utility of storage technologies linked to renewable energy production. Finally, as a mandatory development for commercial deployment, we mention the analysis of global optimization tools capable of providing robust intervals of basic design and operation parameters for all the subsystems composing an actual plant, including, as could not be otherwise, packed-bed subsystems.

## CRediT authorship contribution statement

**D. Pérez-Gallego:** Writing – original draft, Software, Investigation, Conceptualization, Formal analysis, Methodology, Writing – review & editing. **J. Gonzalez-Ayala:** Supervision, Software, Conceptualization, Formal analysis, Investigation, Writing – original draft, Writing – review & editing. **A. Medina:** Writing – review & editing, Writing – original draft, Supervision, Project administration, Conceptualization, Methodology, Visualization. **A. Calvo Hernández:** Writing – review & editing, Supervision, Formal analysis, Funding acquisition, Visualization.

## Declaration of Competing Interest

The authors declare that they have no known competing financial interests or personal relationships that could have appeared to influence the work reported in this paper.

## Figures and Tables

**Table 11:** Properties of some usual phase change materials.

	*NaNO*_2_	Coarser GR50©
*T*_*m*1_ (K)	277.32+273	40+273
*T*_*m*2_ (K)	303.65+273	42+273
*λ*_*l*_ (J/kg)	199600	54379
*c*_*p*,*s*_ (J/(kg⋅K))	1385	1448.6
*c*_*p*,*l*_ (J/(kg⋅K))	1531	1735.7
*ρ*_*s*_ (kg/m^3^)	2097	1512.8
*ρ*_*l*_ (kg/m^3^)	1846	1512.8
*K*_*s*_ (W/(m⋅K))	0.562	0.2
*K*_*l*_ (W/(m⋅K))	0.483	0.2

**Table:** 

Numerical data used in Subsection 6.1			
PB		Walls	carbon steel
*L* [m]	1.0	*e*_*w*_ [mm]	13
*D*_*int*_ [m]	0.7	*ρ*_*w*_ [kg/m^3^]	7840
*ϵ*	0.39	*c*_*p*,*w*_ [J/kgK]	477
Heat transfer fluid	air	*K*_*w*_ [W/mK]	45
Diffuser	yes	Insulation	mineral wool
$\overset{˙}{m}$ [kg/s]	0.5	*e*_*ins*_ [mm]	250
*ρ*_*f*_ [kg/m^3^]	App. E	*ρ*_*ins*_ [kg/m^3^]	32
*c*_*p*,*f*_ [J/kgK]	App. E	*c*_*p*,*ins*_ [J/kgK]	835
*K*_*f*_ [W/mK]	App. E	*K*_*ins*_ [W/mK]	0.038
*μ*_*f*_ [Pa⋅s]	App. E	Boundary conditions	
P${}_{charge}$ [atm]	1.01	Charge: *T*_*f*,*inlet*_ [^*o*^C]	400
Storage material	sand	*T*_*max*_/*T*_*min*_ [^*o*^C]	400/25
*d*_*p*_ [mm]	5-20		
*ρ*_*s*_ [kg/m^3^]	3550		
*c*_*p*,*s*_ [J/kgK]	902		
*K*_*s*_ [W/mK]	0.5		
Emissivity	negligible		

**Table:** 

Numerical data used in PB validation, Section 8				
Data	Cascetta [80]	Izquierdo [108]	Meier (ARIANE) [75]	Xie [73]

PB				
*L* [m]	1.8	0.2	1.2	0.398
*D*_*int*_ [m]	0.584	0.2	0.15	0.194
*ϵ*	0.39	0.473	0.4	0.38

Heat transfer fluid	air	air	air	water
Diffuser	yes	yes	no data	no
$\overset{˙}{m}$ [kg/s]	0.2	0.00777	0.004	0.0083
*Q* [l/min]	12730	450	570	0.5
*ρ*_*f*_ [kg/m^3^]	$2.54676-0.00645271T+6.84089\cdot 10^{-6}T^{2}-2.57974\cdot 10^{-9}T^{3}$			988
*c*_*p*,*f*_ [J/kgK]	$1040.05-0.307614T+0.000743982T^{2}-3.35122\cdot 10^{-7}T^{3}$			4181.3
*K*_*f*_ [W/mK]	$0.00478072+0.0000772337T-1.43807\cdot 10^{-8}T^{2}$			0.64
*μ*_*f*_ [Pa⋅s]	$4.62319\cdot 10^{-6}+5.07777\cdot 10^{-8}T-1.21568\cdot 10^{-11}T^{2}$			5.8⋅10^−4^
P${}_{charge}$ [atm]	1.23	1.01	1.01	1.01

Storage material	alumina beads	GR-50©	magnesium silicate	soda-lime glass
*d*_*p*_ [mm]	8	0.54	20	7
*ρ*_*s*_ [kg/m^3^]	3550	1512.8	2800	2463
*c*_*p*,*s*_ [J/kgK]	−0.0022*T*^2^ + 3.0637*T* + 65.5464	Fig. 19	1095	840
*K*_*s*_ [W/mK]	0.0001*T*^2^ − 0.1773*T* + 79.925	4.0	4.5	1.129
Emissivity	0.8	negligible	negligible	negligible

Walls	carbon steel	stainless steel	stainless steel	transparent polycarbonate
*e*_*w*_ [mm]	13	2	1.5	3
*ρ*_*w*_ [kg/m^3^]	7840	8000	8000	1200
*c*_*p*,*w*_ [J/kgK]	477	500	500	1170
*K*_*w*_ [W/mK]	45	15	15	0.2

**Table:** 

Insulation	mineral wool	glass wool	no data	black nitrile rubber
*e*_*ins*_ [mm]	100	20	no data	25
*ρ*_*ins*_ [kg/m^3^]	32	32	no data	160
*c*_*p*,*ins*_ [J/kgK]	835	835	no data	1350
*K*_*ins*_ [W/mK]	0.038	0.03	no data	0.0412
Boundary conditions				
Charge: *T*_*f*,*inlet*_	Fig. 18(b)	Fig. 20	550 °C	Fig. 21
Discharge: *T*_*f*,*inlet*_	Fig. 18(d)	−−	−−	−−
*T*_*max*_/*T*_*min*_ [^*o*^C]	192/51	65/17	550/25	50/20

## Data Availability

Data will be made available on request. For requesting data, please write to the corresponding author.

## Appendix A Mathematical description of phase change materials and previous approaches to solving the PB model

For completeness purposes, the mathematical details for calculations on PCMs and a summary of the previous studies, including the mathematical models for the resolution of the PB equations, Sec. 3, are presented here.

A temperature range method is used to model the actual transition of latent materials or phase change materials. With this method, two temperatures close to the melting temperature of the material ($T_{m1}$ and $T_{m2}$) are assumed. Below $T_{m1}$, it is considered solid, and the capacity of the solid is applied. Above $T_{m2}$, it is considered liquid and the heat capacity of the liquid is used. In the interval between the two, it is considered that there is a phase transition, and a heat capacity is applied to the material dependent on the latent heat of fusion. Heat capacity density and conductivity of PCM can be written as follows:

(22) $$c_{p,PCM}\left(T\right)=\left\{ \begin{array}{cc} c_{p,s}\; & T<T_{m1},\\ \frac{c_{p,s}+c_{p,l}}{2}+\frac{\lambda_{l}}{T_{m2}-T_{m1}}\; & T_{m1}\leq T\leq T_{m2},\\ c_{p,l}\; & T>T_{m2}, \end{array}\right.$$

(23) $$\rho_{PCM}\left(T\right)=\left\{ \begin{array}{cc} \rho_{s}\; & T<T_{m1},\\ \frac{\rho_{s}+\rho_{l}}{2}\; & \mathrm{si}\;T_{m1}\leq T\leq T_{m2},\\ \rho_{l}\; & T>T_{m2}, \end{array}\right.$$

(24) $$K_{PCM}\left(T\right)=\left\{ \begin{array}{cc} K_{s}\; & T<T_{m1},\\ \frac{K_{s}+K_{l}}{2}\; & \mathrm{si}\;T_{m1}\leq T\leq T_{m2},\\ K_{l}\; & T>T_{m2}. \end{array}\right.$$

In Table 11, there are typical values of $T_{m1}$ and $T_{m2}$ for some PCM. These values are used in Eqs. (22)-(24). However, in this paper, in Subsec. 8.2 to validate Izquierdo et al. [108], a interpolation of $c_{p,s}$ is used (see Fig. 19).

The energy conservation equations of a PB are complex to solve analytically, which is why numerical methods are used. In the case of an analytical resolution, a series of simplifications on the initial equations are required.

A series of simplifications must be used to solve the bed equations analytically. The first of the approaches consists of considering all the magnitudes and properties of the fluid and the bed constant and independent of temperature and position. The second approach consists of neglecting the thermal conductivities in the bed equations. Additionally, to simplify the notation, it includes two dimensionless variables of time and space, defined as follows:

(25) $$\zeta \left(z\right)=\frac{ha_{s}}{\epsilon \rho_{f}c_{p,f}u}\cdot z,$$

(26) $$\tau \left(t\right)=\frac{ha_{s}}{\left(1-\epsilon \right)\rho_{s}c_{p,s}}\cdot t.$$

### A.0.1 Riaz's analytical resolution

Riaz [86] assumes a single-phase approximation, considering that the bed and fluid temperatures are equal at each spatial and temporal instant. In this way, the bed equation for a single-phase model is solved analytically. Also, it is assumed that the heat capacity ($\rho \cdot c_{p}$) of fluid is negligible compared to that of the solid. The energy balance equation can be written as follows:

(27) $$\left[(1-\epsilon )\rho_{s}c_{p,s}\right]\frac{\partial T}{\partial t}+\rho_{f}c_{p,f}\epsilon u\frac{\partial T}{\partial z}=K_{t,eff,z}\frac{\partial^{2}T}{\partial z^{2}}.$$

The temperature profile as a function of time and position is as follows:

(28) $$T\left(t,z\right)=T_{initial}+\left(T_{f,inlet}-T_{initial}\right)\{\frac{1}{2}erfc\left(\frac{\zeta \left(z\right)-\tau \left(t\right)}{2\sqrt{\tau \left(t\right)}}\right)-\frac{1}{2}\left(\zeta \left(z\right)-\tau \left(t\right)\right)e^{\zeta \left(z\right)}erfc\left(\frac{\zeta \left(z\right)-\tau \left(t\right)}{2\sqrt{\tau \left(t\right)}}\right)+\sqrt{\frac{\tau (t)}{\pi }}e^{-\frac{{\left(\zeta \left(z\right)-\tau \left(t\right)\right)}^{2}}{4\tau \left(t\right)}}\},$$

where erfc is the complementary error function.

### A.0.2 Zarty's analytical resolution

Zarty [87] assumes a two-phase model, distinguishing bed temperature from fluid temperature. However, to simplify the equations and obtain an analytical resolution, Zarty assumes a time derivative of the fluid that is negligible compared to the rest of the terms. This approximation is only valid for fluids such as air. The energy balance equation can be written as follows:

(29) $$\epsilon \rho_{f}c_{p,f}u\frac{\partial T_{f}}{\partial z}=ha_{s}(T_{s}-T_{f}),$$

(30) $$(1-\epsilon )\rho_{s}c_{p,s}\frac{\partial T_{s}}{\partial t}=ha_{s}(T_{f}-T_{s}).$$

Or in a dimensionless way:

$$\frac{\partial \theta_{f}}{\partial \zeta }=\theta_{s}-\theta_{f},\frac{\partial \theta_{s}}{\partial \tau }=\theta_{f}-\theta_{s}.$$

The analytical solution can be expressed as follows:

$$T_{f}\left(t,z\right)=T_{f,initial}+\left(T_{f,inlet}-T_{f,initial}\right)e^{-\zeta \left(z\right)}\cdot \int_{0}^{\tau \left(t\right)}e^{-\zeta \left(z\right)-s}I_{1}\left(\sqrt{4s\zeta \left(z\right)}\right)\sqrt{\frac{\zeta \left(z\right)}{s}}ds,T_{s}\left(t,z\right)=T_{s,initial}+\;\left(T_{f,inlet}-T_{s,initial}\right)\int_{0}^{\tau \left(t\right)}e^{-\zeta \left(z\right)-s}I_{0}\left(\sqrt{4s\zeta \left(z\right)}\right)ds,$$

where $I_{0}\left(x\right)$ is the modified Bessel function of the first kind and zero order. $I_{1}\left(x\right)$ is the modified Bessel function of the second kind and first order.

### A.0.3 Duffie's numerical resolution

The importance of numerical resolution compared to analytical consists of the variability of initial and boundary conditions of PB. They can be time or spatial-dependent and not constant, so this causes an impossible problem to solve through analytical solutions. Duffie [88] proposes a *step-response* function, due to exponential charge behavior, to solve Eqs. (29)-(30):

$$T_{f,n}^{t}=T_{s,n-1}^{t}+(T_{f,n-1}^{t}-T_{s,n-1}^{t})e^{-\Delta \zeta \left(z\right)},T_{s,n}^{t}=T_{s,n}^{t-1}+\left(1-e^{-\Delta \zeta \left(z\right)}\right)\left(T_{f,n}^{t-1}-T_{s,n}^{t-1}\right)\frac{\Delta \tau \left(t\right)}{\Delta \zeta \left(z\right)}.$$

## Appendix B Solution to explicit Euler method through iterative algorithm

Below is the solution to the fluid and bed temperatures, for the explicit Euler method through iterative algorithm. For simplicity, the variables A1, B1, C1, D1, E1, A2, B2, C2, are used to express the final solution.

$$A1={\Delta \text{t}}^{2}\Delta \text{z}\;h^{2}a_{s}^{2}\{\Delta \text{z}\left[\rho_{f}c_{p,f}\left(\Delta \text{z}\epsilon T_{f,n}^{t-1}+\Delta \text{t}u\epsilon T_{f,n-1}^{t}\right)+\Delta \text{z}(1-\epsilon )\rho_{s}c_{p,s}T_{s,n}^{t-1}+\Delta \text{t}\Delta \text{z}T_{\infty }Ua_{b}\right]+\Delta \text{t}K_{f}\left(T_{f,n-1}^{t-1}+T_{f,n+1}^{t-1}-2T_{f,n}^{t-1}\right)+\Delta \text{t}K_{s}\left(T_{s,n-1}^{t-1}+T_{s,n+1}^{t-1}-2T_{s,n}^{t-1}\right)\}$$

$$B1=a_{s}h\Delta \text{t}\{\Delta \text{t}\Delta \text{z}Ua_{b}\left[{\Delta \text{z}}^{2}(1-\epsilon )\rho_{s}c_{p,s}T_{s,n}^{t-1}+\Delta \text{t}K_{s}\left(T_{s,n-1}^{t-1}+T_{s,n+1}^{t-1}-2T_{s,n}^{t-1}\right)\right]+\rho_{f}c_{p,f}\{{\Delta \text{z}}^{2}\rho_{s}c_{p,s}\left[\Delta \text{z}(1-\epsilon )\epsilon +u\epsilon (\Delta \text{t}-\Delta \text{t}\epsilon )\right]T_{s,n}^{t-1}+\Delta \text{t}K_{s}\left[(-2\Delta \text{z}\epsilon -2\Delta \text{t}u\epsilon )T_{s,n}^{t-1}+(\Delta \text{z}\epsilon +\Delta \text{t}u\epsilon )\left(T_{s,n-1}^{t-1}+T_{s,n+1}^{t-1}\right)\right]\}\}$$

$$C1=\Delta \text{t}ha_{s}\left[\rho_{f}c_{p,f}(\Delta \text{z}\epsilon +\Delta \text{t}u\epsilon )+\Delta \text{t}\Delta \text{z}Ua_{b}\right]+(1-\epsilon )\rho_{s}c_{p,s}\left[\rho_{f}c_{p,f}(\Delta \text{z}\epsilon +\Delta \text{t}u\epsilon )+\Delta \text{t}\Delta \text{z}Ua_{b}+\Delta \text{t}\Delta \text{z}ha_{s}\right]$$

$$D1={\Delta \text{z}}^{2}\epsilon \rho_{f}c_{p,f}T_{f,n}^{t-1}+\Delta \text{t}\Delta \text{z}\text{Us}\rho_{f}c_{p,f}T_{f,n-1}^{t}+\Delta \text{t}K_{f}T_{f,n-1}^{t-1}-2\Delta \text{t}K_{f}T_{f,n}^{t-1}+\Delta \text{t}K_{f}T_{f,n+1}^{t-1}+\Delta \text{t}{\Delta \text{z}}^{2}T_{\infty }Ua_{b}$$

$$E1=\Delta \text{z}\left(\rho_{f}c_{p,f}(\Delta \text{z}\epsilon +\Delta \text{t}u\epsilon )+\Delta \text{t}\Delta \text{z}Ua_{b}+\Delta \text{t}\Delta \text{z}ha_{s}\right)$$

$$T_{f,n}^{t}=\frac{\frac{A1+B1}{C1}+D1}{E1}$$

$$A2=\Delta \text{t}\Delta \text{z}ha_{s}\{\Delta \text{z}\left[\rho_{f}c_{p,f}\left(\Delta \text{z}\epsilon T_{f,n}^{t-1}+\Delta \text{t}u\epsilon T_{f,n-1}^{t}\right)+\Delta \text{z}(1-\epsilon )\rho_{s}c_{p,s}T_{s,n}^{t-1}+\Delta \text{t}\Delta \text{z}T_{\infty }Ua_{b}\right]+\Delta \text{t}K_{f}\left(T_{f,n-1}^{t-1}+T_{f,n+1}^{t-1}-2T_{f,n}^{t-1}\right)+\Delta \text{t}K_{s}\left(T_{s,n-1}^{t-1}+T_{s,n+1}^{t-1}-2T_{s,n}^{t-1}\right)\}$$

$$B2=\Delta \text{t}\Delta \text{z}Ua_{b}\left[{\Delta \text{z}}^{2}(1-\epsilon )\rho_{s}c_{p,s}T_{s,n}^{t-1}+\Delta \text{t}K_{s}\left(T_{s,n-1}^{t-1}+T_{s,n+1}^{t-1}-2T_{s,n}^{t-1}\right)\right]+\rho_{f}c_{p,f}\{{\Delta \text{z}}^{2}\rho_{s}c_{p,s}\left[\Delta \text{z}(1-\epsilon )\epsilon +u\epsilon (\Delta \text{t}-\Delta \text{t}\epsilon )\right]T_{s,n}^{t-1}+\Delta \text{t}K_{s}\left[(-2\Delta \text{z}\epsilon -2\Delta \text{t}u\epsilon )T_{s,n}^{t-1}+(\Delta \text{z}\epsilon +\Delta \text{t}u\epsilon )\left(T_{s,n-1}^{t-1}+T_{s,n+1}^{t-1}\right)\right]\}$$

$$C2={\Delta \text{z}}^{2}\{\Delta \text{t}\Delta \text{z}Ua_{b}\left((1-\epsilon )\rho_{s}c_{p,s}+\Delta \text{t}ha_{s}\right)+\Delta \text{t}ha_{s}\left(\rho_{f}c_{p,f}(\Delta \text{z}\epsilon +\Delta \text{t}u\epsilon )+\Delta \text{z}(1-\epsilon )\rho_{s}c_{p,s}\right)+\rho_{f}\rho_{s}c_{p,f}c_{p,s}\left[\Delta \text{z}(1-\epsilon )\epsilon +u\epsilon (\Delta \text{t}-\Delta \text{t}\epsilon )\right]\}$$

$$T_{s,n}^{t}=\frac{A2+B2}{C2}$$

## Appendix C Matrix used in implicit Euler method

Next, the arrays of the implicit Euler method through the matrix algorithm are presented. Equation (14) is solved using these arrays, so temperatures of fluid and bed can be obtained. The arrays are built using Eqs. (12)-(13) and (15)-(16).

Where:

$$\alpha =\frac{\epsilon \rho_{f}c_{p,f}}{\Delta t}+\epsilon \rho_{f}c_{p,f}\frac{u}{\Delta z}+\frac{2\epsilon K_{f,eff,z}}{\Delta z^{2}}+h_{eff}a_{s}+Ua_{b}\;\beta =-\epsilon \rho_{f}c_{p,f}\frac{u}{\Delta z}-\frac{\epsilon K_{f,eff,z}}{\Delta z^{2}}\;\gamma =-\frac{\epsilon K_{f,eff,z}}{\Delta z^{2}}\;\delta =-h_{eff}a_{s}\;\epsilon =\frac{\epsilon \rho_{f}c_{p,f}}{\Delta t}\;\eta =Ua_{b}T_{\infty }\;\alpha^{\prime }=\frac{\left(1-\epsilon \right)\rho_{s}c_{p,s}}{\Delta t}+\frac{2\left(1-\epsilon \right)K_{\text{s,eff,z}}}{\Delta z^{2}}+h_{eff}a_{s}\;\beta^{\prime }=-\frac{\left(1-\epsilon \right)K_{\text{s,eff,z}}}{\Delta z^{2}}\;\gamma^{\prime }=-\frac{\left(1-\epsilon \right)K_{\text{s,eff,z}}}{\Delta z^{2}}\;\delta^{\prime }=-h_{eff}a_{s}\;\epsilon^{\prime }=\frac{\left(1-\epsilon \right)\rho_{s}c_{p,s}}{\Delta t}$$

## Appendix D Numerical data for overview between models

In this section, the numerical data used for the overview between models are shown.

## Appendix E Test rig data for validations

In this section, the properties and data for validation of test rigs are presented.

## References

[1] (2021). http://www.irena.org/publications.

[2] Olympios A.V., McTigue J.D., Farres-Antunez P., Tafone A., Romagnoli A., Li Y., Ding Y., Steinmann W.D., Wang L., Chen H., Markides C.N. (2021). Progress and prospects of thermo-mechanical energy storage: a critical review. Prog. Energy.

[3] Rahman M.M., Oni A.O., Gemechu E., Kumar A. (2020). Assessment of energy storage technologies: a review. Energy Convers. Manag..

[4] Bartela L., Ochmann J., Waniczek S., Lutyński M., Smolnik G., Rulik S. (2022). Evaluation of the energy potential of an adiabatic compressed air energy storage system based on a novel thermal energy storage system in a post mining shaft. J. Energy Storage.

[5] Vecchi A., Li Y., Ding Y., Mancarella P., Sciacovelli A. (2021). Liquid air energy storage (LAES): a review on technology state-of-the-art, integration pathways and future perspectives. Adv. Appl. Energy.

[6] Schäppi R., Rutz D., Dähler F., Muroyama A., Haueter P., Liliestam J., Patt A., Furler P., Steinfeld A. (2021). Drop-in fuels from sunlight and air. Nature.

[7] Carrillo A.J., González-Aguilar J., Romero M., Coronado J.M. (2019). Solar energy on demand: a review on high temperature thermochemical heat storage systems and materials. Chem. Rev..

[8] Benato A., Stoppato A. (2018). Pumped thermal electricity storage: a technology overview. Therm. Sci. Eng. Prog..

[9] Achkari O., El Fadar A. (2020). Latest developments on TES and CSP technologies-energy and environmental issues, applications and research trends. Appl. Therm. Eng..

[10] Steinmann W.D., Jockenhöfer H., Bauer D. (2020). Thermodynamic analysis of high-temperature Carnot battery concepts. Energy Technol..

[11] Gallo A.B., Moreira J.R. Simões, Costa H.K.M., Santos M.M., Santos E. Moutinho dos (2016). Energy storage in the energy transition context: a technology review. Renew. Sustain. Energy Rev..

[12] (2020). http://www.irena.org/publications.

[13] McTigue J.D., White A.J., Markides C.N. (2015). Parametric studies and optimisation of pumped thermal electricity storage. Appl. Energy.

[14] McTigue J.D., Farres-Antunez P., Sundarnath K., Markides C.N., White A.J. (2022). Techno-economic analysis of recuperated Joule-Brayton pumped thermal energy storage. Energy Convers. Manag..

[15] Khan M.I., Asfand F., Al-Ghamdi S.G. (2022). Progress in research and technological advancements of thermal energy storage systems for concentrated solar power. J. Energy Storage.

[16] Kocak B., Paksoy H. (2020). Performance of laboratory scale packed-bed thermal energy storage using new demolition waste based sensible heat materials for industrial solar applications. Sol. Energy.

[17] Schumann T.E.W. (1929). Heat transfer: a liquid flowing through a porous prism. J. Franklin Inst..

[18] Al-Nimr M., Abu-Qudais M.K., Mashqai M.D. (1996). Dynamic behavior of a packed bed energy storage system. Energy Convers. Manag..

[19] Baigorri J., Zaversky F., Astrain D. (2023). Massive grid-scale energy storage for next-generation concentrated solar power: a review of the potential emerging concepts. Renew. Sustain. Energy Rev..

[20] Calderón-Vásquez I., Cortés E., García J., Segovia V., Caroca A., Sarmiento C., Barraza R., Cardemil J.M. (2021). Review on modeling approaches for packed-bed thermal storage systems. Renew. Sustain. Energy Rev..

[21] Zurita A., Mata-Torres C., Cardemil J.M., Escobar R.A. (2020). Assessment of time resolution impact on the modeling of a hybrid CSP-PV plant: a case of study in Chile. Sol. Energy.

[22] Ellingwood K., Safdarnejad S., Kovacs H., Tuttle J., Powell K. (2019). Analysing the benefits of hybridisation and storage in a hybrid solar gas turbine plant. Int. J. Sustain. Energy.

[23] Merchán R.P., Santos M.J., Medina A., Calvo Hernández A. (2022). High temperature central tower plants for concentrated solar power: 2021 overview. Renew. Sustain. Energy Rev..

[24] González I., Pérez-Segarra C.D., Lehmkuhl O., Torras S., Oliva A. (2016). Thermo-mechanical parametric analysis of packed-bed thermocline energy storage tanks. Appl. Energy.

[25] Petrollese M., Cascetta M., Cocco D., Tola V., Cau G. (2021). Proceedings of ECOS 2021, ECOS 2021.

[26] Petrollese M., Cascetta M., Tola V., Cocco D., Cau G. (2022). Pumped thermal energy storage systems integrated with a concentrating solar power section: conceptual design and performance evaluation. Energy.

[27] Alva G., Liu L., Huang X., Fang G. (2017). Thermal energy storage materials and systems for solar energy applications. Renew. Sustain. Energy Rev..

[28] Gautam A., Saini R.P. (2020). A review on technical, applications and economic aspect of packed bed solar thermal energy storage system. J. Energy Storage.

[29] Trevisan S., Jemmal Y., Guedez R., Laumert B. (2021). Packed bed thermal energy storage: a novel design methodology including quasi-dynamic boundary conditions and techno-economic optimization. J. Energy Storage.

[30] Liu M., Steven Tay N.H., Bell S., Belusko M., Jacob R., Will G., Saman W., Bruno F. (2016). Review on concentrating solar power plants and new developments in high temperature thermal energy storage technologies. Renew. Sustain. Energy Rev..

[31] Palacios A., Barreneche C., Navarro M., Ding Y. (2020). Thermal energy storage technologies for concentrated solar power-a review from a materials perspective. Renew. Energy.

[32] Pelay U., Luo L., Fan Y., Stitou D., Rood M. (2017). Thermal energy storage systems for concentrated solar power plants. Renew. Sustain. Energy Rev..

[33] Suresh C., Saini R.P. (2019). Review on solar thermal energy storage technologies and their geometrical configurations. Int. J. Energy Res..

[34] Trevisan S., Wang W., Laumert B. (2021). Coatings utilization to modify the effective properties of high temperature packed bed thermal energy storage. Appl. Therm. Eng..

[35] Dumont O., Frate G.F., Pillai A., Lecompte S., de Paepe M., Lemort V. (2020). Carnot battery technology: a state-of-the-art review. J. Energy Storage.

[36] Sharma S., Mortazavi M. (2023). Pumped thermal energy storage: a review. Int. J. Heat Mass Transf..

[37] Salomone-González D., Curto-Risso P.L., Calvo Hernández A., Medina A., Roco J.M.M., González-Ayala J. (2022). Pumped heat energy storage with liquid media: thermodynamic assessment by a transcritical rankine-like model. J. Energy Storage.

[38] Pérez-Gallego D., Gonzalez-Ayala J., Calvo Hernández A., Medina A. (2021). Thermodynamic performance of a Brayton pumped heat energy storage system: influence of internal and external irreversibilities. Entropy.

[39] Fischer D., Madani H. (2017). On heat pumps in smart grids: a review. Renew. Sustain. Energy Rev..

[40] Salomone-González D., González-Ayala J., Medina A., Roco J.M.M., Curto-Risso P.L., Calvo Hernández A. (2020). Pumped heat energy storage with liquid media: thermodynamic assessment by a Brayton-like model. Energy Convers. Manag..

[41] Ge Y.Q., Zhao Y., Zhao C.Y. (2021). Transient simulation and thermodynamic analysis of pumped thermal electricity storage based on packed-bed latent heat/cold stores. Renew. Energy.

[42] (Jan. 2019). https://www.theengineer.co.uk/content/news/newcastle-university-connects-first-grid-scale-pumped-heat-energy-storage-system.

[43] Ameen M.T., Ma Z., Smallbone A., Norman R., Roskilly A.P. (2023). Demonstration system of pumped heat energy storage (phes) and its round-trip efficiency. Appl. Energy.

[44] Smallbone A., Jülch V., Wardle R., Roskilly A.P. (2017). Levelised cost of storage for pumped heat energy storage in comparison with other energy storage technologies. Energy Convers. Manag..

[45] Davenne T.R., Peters B.M. (2020). An analysis of pumped thermal energy storage with de-coupled thermal stores. Front. Energy Res..

[46] Benato A. (2017). Performance and cost evaluation of an innovative pumped thermal electricity storage power system. Energy.

[47] Zhao Y., Song J., Liu M., Zhao Y., Olympos A.V., Sapin P., Yan J., Markides C.N. (2022). Thermo-economic assessments of pumped-thermal electricity storage systems employing sensible heat storage materials. Renew. Energy.

[48] Laughlin R.B. (2017). Pumped thermal grid storage with heat exchange. J. Renew. Sustain. Energy Rev..

[49] Farres-Antunez P., McTigue J.D., White A.J. (2019). Proceedings of the 2019 Offshore Energy and Storage Summit, OSES 2019, Offshore Energy and Storage Summit.

[50] Cascetta M., Licheri F., Merchán R.P., Petrollese M. (2023). Operating performance of a Joule-Brayton pumped thermal energy storage system integrated with a concentrated solar power plant. J. Energy Storage.

[51] Merchán R.P., Petrollese M., Cau G. (2023). Proceedings of ECOS, ECOS 2023.

[52] Bazdar E., Sameti M., Nasiri F., Haghighat F. (2022). Compressed air energy storage in integrated energy systems: a review. Renew. Sustain. Energy Rev..

[53] Zhou Q., Du D., Lu C., He Q., Liu W. (2019). A review of thermal energy storage in compressed air energy storage system. Energy.

[54] Zunft S., Dreissigacker V., Bieber M., Banach A., Klabunde C., Warweg O. (2017).

[55] Tong Z., Cheng Z., Tong S. (2021). A review on the development of compressed air energy storage in China: technical and economic challenges to commercialization. Renew. Sustain. Energy Rev..

[56] Barbour E.R., Pottie D.L., Eames P. (2021). Why is adiabatic compressed air energy storage yet to become a viable energy storage option?. iScience.

[57] Barbour E., Pottie D.L. (2021). Adiabatic compressed air energy storage technology. Joule.

[58] Tola V., Meloni V., Spadaccini F., Cau G. (2017). Performance assessment of adiabatic compressed air energy storage (ACAES) power plants integrated with packed-bed thermocline storage systems. Energy Convers. Manag..

[59] Morgan R., Nelmes S., Gibson E., Brett G. (2015).

[60] Morgan R., Nelmes S., Gibson E., Brett G. (2015). Liquid air energy storage - analysis and first results from a pilot scale demonstration plant. Appl. Energy.

[61] (Oct. 2019). https://highviewpower.com/news_announcement/highview-power-to-develop-multiple-cryogenic-energy-storage-facilities-in-the-uk-and-to-build-europes-largest-storage-system/.

[62] Singh H., Saini R.P., Saini J.S. (2010). A review on packed bed solar energy storage systems. Renew. Sustain. Energy Rev..

[63] Bespalko S., Munoz Miranda A., Halychyi O. (2018). Overview of the existing heat storage technologies: sensible heat. Acta Innov..

[64] Ameen M.T., Ma Z., Smallbone A., Norman R., Norman R., Roskilly A.P. (2023). Experimental study and analysis of a novel layered packed-bed for thermal energy storage applications: a proof of concept. Energy Convers. Manag..

[65] Pielichowska K., Pielichowski K. (2014). Phase change materials for thermal energy storage. Prog. Mater. Sci..

[66] Haynes W.M. (2016).

[67] Green D.W., Southard M. (2007).

[68] Schröder J. (1975). Thermal energy storage and control. J. Eng. Ind..

[69] Zalba B., Marín J.M., Cabeza L.F., Mehling H. (2003). Review on thermal energy storage with phase change: materials, heat transfer analysis and applications. Appl. Therm. Eng..

[70] O'Neil M.J. (2001).

[71] Cascetta M., Cau G., Puddu P., Serra F. (2014). Numerical investigation of a packed bed thermal energy storage system with different heat transfer fluids. Energy Proc..

[72] Esence T., Bruch A., Fourmigue J.F., Stutz B. (2019). A versatile one-dimensional numerical model for packed-bed heat storage systems. Renew. Energy.

[73] Xie B., Baudin N., Soto J., Fan Y., Luo L. (2023). Experimental and numerical study onfillers. Renew. Energy.

[74] Douglas M. (1986).

[75] Meier A., Winkler C., Wuillemin D. (1991). Experiment for modelling high temperature rock bed storage. Sol. Energy Mater..

[76] Pacheco J.E., Showalter S.K., Kolb W.J. (2002). Development of a molten-salt thermocline thermal storage system for parabolic trough plant. J. Sol. Energy Eng..

[77] Yang X., Ding J., Shao Y., Qin G.G., Jiang R. (2012). Criteria for performance improvement of a molten salt thermocline storage system. Appl. Therm. Eng..

[78] Anderson R., Shiri H., Bindra S., Morris J.F. (2014). Experimental results and modeling of energy storage and recovery in a packed bed of alumina particles. Appl. Energy.

[79] Bruch A., Fourmigué J.F., Couturier R. (2014). Experimental and numerical investigation of a pilot-scale thermal oil packed bed thermal storage system for CSP power plant. Sol. Energy.

[80] Cascetta M., Cau G., Puddu P., Serra F. (2016). A comparison between CFD simulation and experimental investigation of a packed-bed thermal energy storage system. Appl. Therm. Eng..

[81] Hoffmann J.F., Fasquelle T., Goetz V., Py X. (2017). Experimental and numerical investigation of a thermocline thermal energy storage tank. Appl. Therm. Eng..

[82] Esence T., Desrues T., Fourmigué J.F., Cwicklinski G., Bruch A., Stutz B. (2019). Experimental study and numerical modelling of high temperature gas/solid packed-bed heat storage systems. Energy.

[83] Keilany M.A., Milhé M., Bézian J.-J., Falcoz Q., Flamant G. (2020). Experimental evaluation of vitrified waste as solid fillers used in thermocline thermal energy storage with parametric analysis. J. Energy Storage.

[84] Vannerem S., Neveu P., Falcoz Q. (2021). Experimental and numerical investigation of the impact of operating conditions on thermocline storage performance. Renew. Energy.

[85] Xu C., Liu M., Jiao S., Tang H., Yan J. (2022). Experimental study and analytical modeling on the thermocline hot water storage tank with radial plate-type diffuser. Int. J. Heat Mass Transf..

[86] Riaz M. (1978). Transient analysis of packed-bed thermal storage systems. Sol. Energy.

[87] Zarty A., Juddaimi A.E. (1987). Computational models of a rock-bed thermal storage unit. Solar Wind Technol..

[88] Duffie J., Beckman W. (2006). https://onlinelibrary.wiley.com/doi/pdf/10.1002/9781118671603.

[89] Bindra H., Bueno P., Morris J.F., Shinnar R. (2013). Thermal analysis and exergy evaluation of packed bed thermal storage systems. Appl. Therm. Eng..

[90] Rosen M.A. (2001). The exergy of stratified thermal energy storages. Sol. Energy.

[91] Li G. (2016). Sensible heat thermal storage energy and exergy performance evaluations. Renew. Sustain. Energy Rev..

[92] Calderón-Vásquez I., Cardemil J.M. (2024). A comparison of packed-bed flow topologies for high-temperature thermal energy storage under constrained conditions. Appl. Therm. Eng..

[93] Barbour E., Mignard D., Ding Y., Li Y. (2015). Adiabatic compressed air energy storage with packed bed thermal energy storage. Appl. Energy.

[94] Geissbühler L., Becattini V., Zanganeh G., Zavattoni S., Barbato M., Haselbacher A., Steinfeld A. (2018). Pilot-scale demonstration of advanced adiabatic compressed air energy storage, part 1: plant description and tests with sensible thermal-energy storage. J. Energy Storage.

[95] Wakao N., Kaguei S., Funazkr T. (1979). Effect of fluid dispersion coefficients on particle-to-fluid heat transfer coefficients in packed beds. Chem. Eng. Sci..

[96] Beek J. (1962). Design of packed catalytic reactors. Adv. Chem. Eng..

[97] Ismail K.A.R., Stuginsky R. (1999). A parametric study on possible fixed bed models for PCM and sensible heat storage. Appl. Therm. Eng..

[98] Esence T., Bruch A., Molina S., Stutz B., Fourmigué J.F. (2017). A review on experience feedback and numerical modeling of packed-bed thermal energy storage systems. Sol. Energy.

[99] Xie B., Baudin N., Soto J., Fan Y., Luo L. (2022). Wall impact on efficiency of packed-bed thermocline thermal energy storage system. Energy.

[100] Churchill S.W., Chu H.H.S. (1975). Correlating equations for laminar and turbulent free convection from a vertical plate. Int. J. Heat Mass Transf..

[101] Bergman T., Lavine A., Incropera F., Dewitt D. (2012).

[102] Wakao N., Kagei S. (1982).

[103] Lienhard J.H., Lienhard J.H. (2024). https://ahtt.mit.edu.

[104] COMSOL Multiphysics COMSOL. https://doc.comsol.com/6.3/docserver/#!/com.comsol.help.comsol/helpdesk/helpdesk.html.

[105] Elouali A., Kousksou T., El Rhakifi T., Hamdaoui S., Mahdaoui M., Allouhi A., Zeraouli Y. (2019). Physical models for packed bed: sensible heat storage systems. J. Energy Storage.

[106] Odenthal C., Klasing F., Bauer T. (2019). A three-equation thermocline thermal energy storage model for bidisperse packed beds. Sol. Energy.

[107] Esence T., Bruch A., Fourmigué J.F., Stutz B. (2018).

[108] Izquierdo-Barrientos M.A., Sobrino C., Almendros-Ibáñez J.A. (2013). Thermal energy storage in a fluidized bed of PCM. Chem. Eng. J..

[109] Whitaker S. (1986). Flow in porous media I: a theoretical derivation of Darcy's law. Transp. Porous Media.

[110] Kaviany M. (2012).

[111] Ergun S., Orning A.A. (1949). Fluid flow through randomly packed columns and fluidized beds. Ind. Eng. Chem..

[112] Peng H., Dong H., Ling X. (2014). Thermal investigation of PCM-based high temperature thermal energy storage in packed bed. Energy Convers. Manag..

[113] Trevisan S., Guedez R. (2024). Design optimization of an innovative layered radial-flow high-temperature packed bed thermal energy storage. J. Energy Storage.

